# On a path toward a broad-spectrum anti-viral: inhibition of HIV-1 and coronavirus replication by SR kinase inhibitor harmine

**DOI:** 10.1128/jvi.00396-23

**Published:** 2023-09-14

**Authors:** Subha Dahal, Kiera Clayton, Tyler Cabral, Ran Cheng, Shahrzad Jahanshahi, Choudhary Ahmed, Amrit Koirala, Alonso Villasmil Ocando, Ramy Malty, Terek Been, Javier Hernandez, Maria Mangos, David Shen, Mohan Babu, John Calarco, Benoit Chabot, Liliana Attisano, Walid A. Houry, Alan Cochrane

**Affiliations:** 1 Department of Molecular Genetics, University of Toronto, Toronto, Ontario, Canada; 2 Department of Pathology, University of Massachusetts Medical School, Worcester, Massachusetts, USA; 3 Department of Biochemistry, University of Toronto, Toronto, Ontario, Canada; 4 Department of Molecular and Cellular Biology, Baylor College of Medicine, Houston, Texas, USA; 5 Dan L. Duncan Cancer Comprehensive Center, Baylor College of Medicine, Houston, Texas, USA; 6 Ragon Institute of MGH, MIT and Harvard, Cambridge, Massachusetts, USA; 7 Research and Innovation Centre, Department of Biochemistry, University of Regina, Regina, Saskatchewan, Canada; 8 Department of Cell and Systems Biology, University of Toronto, Toronto, Ontario, Canada; 9 Department of Microbiology and Infectious Diseases, Université de Sherbrooke, Sherbrooke, Quebec, Canada; 10 Department of Chemistry, University of Toronto, Toronto, Ontario, Canada; Icahn School of Medicine at Mount Sinai, New York, New York, USA

**Keywords:** HIV-1, coronaviruses, RNA processing, SR kinase, inhibitors

## Abstract

RNA processing plays a key role in gene expression, allowing for increased protein diversity and functional complexity. Consequently, modulating RNA processing can impact gene function. Given HIV-1’s reliance on host RNA processing machinery for viral protein production/replication, modulators of this process could serve as novel anti-virals to complement and/or enhance existing therapies. In this study, screening of several serine-arginine-rich (SR) kinase inhibitors for their impact on HIV-1 gene expression identified harmine as an inhibitor of HIV-1 gene expression in several cell lines and primary CD4^+^ T cells/macrophages at low micromolar concentrations with limited cell toxicity. Harmine induced a loss of viral structural protein expression associated with reduced HIV-1 unspliced and singly-spliced HIV-1 RNA levels but limited impact on multiply spliced RNAs. Although harmine is a known inhibitor of both DYRK1A and monoaminoxidase A (MAO A), neither DYRK1A depletion nor other MAO A inhibitors had any effect on HIV-1 expression. However, the compound altered the expression of several other SR kinases in primary CD4^+^ T cells, increasing CLK1 and reducing CLK2 kinase levels, effects known to regulate HIV-1 expression. Harmine was also unique among the SR kinase inhibitors tested for its ability to suppress replication of a seasonal coronavirus, human coronavirus (HCoV)-229E, and multiple severe acute respiratory syndrome coronavirus 2 (SARS-CoV-2) variants, reducing viral protein expression and virus release. Harmine acts post-entry, arresting virus replication even after the onset of viral protein production. At doses required to suppress HIV-1 replication, harmine had limited impact on the host transcriptome, alternative splicing, or alterative polyadenylation as assessed by RNA-Seq. Together, our study demonstrates the feasibility of targeting host RNA processing to inhibit a range of viruses with minimal impact on the host cell.

**IMPORTANCE:**

This study highlights the crucial role RNA processing plays in regulating viral gene expression and replication. By targeting SR kinases, we identified harmine as a potent inhibitor of HIV-1 as well as coronavirus (HCoV-229E and multiple SARS-CoV-2 variants) replication. Harmine inhibits HIV-1 protein expression and reduces accumulation of HIV-1 RNAs in both cell lines and primary CD4^+^ T cells. Harmine also suppresses coronavirus replication post-viral entry by preferentially reducing coronavirus sub-genomic RNA accumulation. By focusing on host factors rather than viral targets, our study offers a novel approach to combating viral infections that is effective against a range of unrelated viruses. Moreover, at doses required to inhibit virus replication, harmine had limited toxicity and minimal effect on the host transcriptome. These findings support the viability of targeting host cellular processes as a means of developing broad-spectrum anti-virals.

## INTRODUCTION

Due to their limited genome size, viruses depend on multiple host cell functions for gene expression and virion assembly/release (1). If viruses from diverse taxonomic groups are dependent on the same cellular process, modulating the process offers an alternative strategy to inhibit virus replication and generate broad-spectrum anti-virals (1). Many viruses exploit alternative RNA splicing to expand their coding capacity, rendering them sensitive to splicing modulators (2 – 5). In addition to the core components of the host splicing apparatus, the serine-arginine-rich (SR) family of proteins plays an important role in the regulation of gene expression by controlling constitutive and alternative splicing (AS) of RNAs (6). Besides their known roles in spliceosome assembly and splice-site recognition (7), SR proteins also have been implicated in the regulation of transcription, polyadenylation, export of mRNA, RNA stability, and translation (6, 8). The activity and localization of SR proteins are controlled by their phosphorylation by a variety of SR kinases (9, 10) including serine/arginine-specific protein kinases (SRPKs), Cdc2-like kinases (CLKs), and dual-specificity tyrosine-regulated kinases (DYRKs), all sharing the common property of phosphorylating SR proteins at the serine residues within their RS (arginine-serine rich) region (7, 11, 12). Several studies have demonstrated that SR kinases have a role in regulating viral replication via direct or indirect mechanisms (phosphorylation/dephosphorylation of cellular and/or viral targets, cellular response to viral infection) (5, 6, 13, 14). Interestingly, SR kinases and their substrates influence not only viruses that replicate in the nucleus [HIV-1, influenza, and adenovirus (5, 13)] but also many RNA viruses whose entire life cycle occurs in the cytoplasm (14, 15). Consequently, in search of potential broad-spectrum anti-virals, we examined the effect of several SR kinase inhibitors on the replication of both HIV-1 and coronaviruses.

Production of the full spectrum of proteins encoded by HIV-1 critically relies on alternative splicing events involving cellular splicing factors, including the SR proteins (2) and the kinases that phosphorylate them (13, 16). To examine HIV-1’s dependence on host AS for its replication (2), we tested several small molecule SR kinase inhibitors (leucettine 41 (L41), TG003, KH-CB19, and harmine) (17, 18) for their ability to inhibit HIV-1 gene expression. Using HeLa and T cell lines, we identified that, of the compounds tested, only harmine inhibited HIV-1 protein expression with minimal cytotoxicity. Reduced viral protein expression upon harmine addition was associated with altered viral mRNA accumulation. Harmine also reduced viral protein levels and altered accumulation of viral RNAs in primary CD4^+^ T cells infected with the HIV-1_89.6_ strain. In the context of infected macrophages, the compound had limited impact on percent infection but reduced Gag expression levels in infected cells post-integration.

In parallel, we tested the same panel of SR kinase inhibitors for their ability to inhibit seasonal coronavirus, human coronavirus (HCoV)-229E, and again identified harmine as a potent inhibitor. Harmine treatment of HCoV-229E or severe acute respiratory syndrome coronavirus 2 (SARS-CoV-2)-infected cells reduced viral protein and RNA accumulation, resulting in suppression of virus release. Harmine retained its ability to suppress virus growth even 16 h after addition of virus inoculum, indicating that it affects post-entry steps of the coronavirus life cycle. To decipher the mechanism underlying harmine’s anti-HIV-1 activity, we explored the role of its known targets, SR kinase DYRK1A and monoamine oxidase A (MAO A) (18) in the control of HIV-1 gene expression. Neither DYRK1A depletion nor addition of other MAO A inhibitors affected HIV-1 gene expression, indicating that harmine’s effect on HIV-1 is mediated through other proteins/pathways. Interestingly, harmine-induced changes in SR protein expression/phosphorylation were associated with a dramatic increase in CLK1 levels and a reduction in CLK2 levels consistent with our recent studies demonstrating that depletion of CLK2 blocks HIV-1 gene expression (16). These findings suggest that harmine’s anti-viral effect could be mediated through changes in the relative abundance of CLK1 or CLK2 levels. Harmine also altered the abundance of select SR proteins known to play important roles in HIV-1 RNA processing (19). At the dose used to inhibit HIV-1, harmine had very limited impact on the global cellular transcriptome of CD4^+^ T cells as measured by changes in gene expression, alternative splicing, and alternative polyadenylation (APA) usage, suggesting that the viruses are very sensitive to the effects of this compound. Together, the findings presented in our study support the potential of targeting host RNA processing factors for complementary and/or alternative therapeutic approaches against different viral infections.

## MATERIALS AND METHODS

### Cell lines, primary cells, lung organoids, and virus strains

#### Cell lines

HeLa B2 (HeLa rtTA HIV∆mls) cells were used to screen/test compounds for their effect on HIV as previously described (13). The CEM-HIV cell line generated by transduction of CEM-CD4^+^ T cells (NIH AIDS Reagent Program Cat #117, Bethesda, MD, USA) with the proviral construct, HIV rtTA GagzipGFP, has been recently described (16). HEK 293 cells for lentivirus production were cultured in Eagle’s minimum essential medium (MEM; Gibco, Waltham, MA, USA) supplemented with 10% fetal bovine serum (FBS) (Sigma-Aldrich, St. Louis, MO, USA) plus penicillin (100 U/mL) and streptomycin (100 µg/mL). Huh7 cells used for infection with coronaviruses were maintained and used as previously described (5). All the cell lines were maintained at 37°C in a humidified incubator with 5% CO_2_.

#### Primary cells

Macrophages and CD4^+^ T cells were prepared as previously described (19, 20). Buffy coats from anonymous healthy donors (HIV uninfected) were acquired from the Massachusetts General Hospital Blood Bank.

#### Generation of human lung organoids

Human lung organoids were generated in the Applied Organoid Core Facility (Donnelly Centre, University of Toronto) with a modified protocol (21) using H9 human embryonic stem cells (provided by James A. Thomson, University of Wisconsin-Madison to the WiCell Research Institute). In brief, H9s plated in V-bottom plates were differentiated into endoderm in DMEM/F12 (Life Technologies), 20% KnockOut Serum Replacement (Life Technologies), 2% MEM- non-essential amino acids solution (NMEM-NEAA; Gibco, cat # 11140-050), 55-µM β-mercaptoethanol (Life Technologies), 50-µM Y-27632 (Selleck Chemicals, cat # S1049) using 3-mM CHIR99021 (Sellleck Chem. #S1263), 100-ng/mL activin A. For induction of anterior foregut endoderm, cells were transferred to serum-free media (SFM: DMEM/F12 [HAM], with N2, B27 with vitamin A, ascorbic acid, GlutaMAX, and N-acetyl-L-cysteine) supplemented with 300-nM low-dose naltrexone (LDN) and 10-µM SB431542 for 24 h and then to SFM supplemented with 1-µM IWP-2 and 10-µM SB431542 for another 24 h. To induce lung progenitors, cells were incubated with SFM supplemented with SAG, CHIR, BMP4, and all-*trans* retinoic acid for 2 days. The early lung organoids were then embedded in Matrigel in maturation media composed of SFM supplemented with CHIR, FGF7, FGF10, BMP4, and all-*trans* retinoic acid for 7–10 days with media changes every 3 days. Lung organoids were then excised and maintained as air-liquid interface cultures until day 30.

#### Virus strains

HIV-1_89.6_ (an R5 × 4 dual-tropic primary isolate of HIV-1) virus production has been previously described (16, 20). Human coronavirus -229E was obtained from American Type Culture Collection (ATCC, Manassas, VA, USA). SB2, Delta, and Omicron BA.1 variants of SARS-CoV-2 were obtained from Dr. S. Mubareka (University of Toronto, Toronto, ON, Canada) and the combined Containment Level 3 unit at the University of Toronto.

### shRNA plasmids and lentivirus production

Plasmids expressing shRNAs targeting DYRK1A were generously provided by Dr. Jason Moffat (University of Toronto). The shRNA sequences were cloned into pLKO.1 expression vector that drives shRNA expression from a human U6 promoter and contains a puromycin selection marker (21). shRNAs for depletion of DYRK1A targeted either CAGTATATTCAGAGTCGCTTT or CGGAAGGTTTACAATGATGGT. To generate shRNA lentiviruses, HEK293T17 cells (Cat# ATCC CRL-11268) were transfected with three-plasmid lentiviral packaging system comprising pLKO shRNA vector (6 µg), vesicular stomatitis virus G protein envelope plasmid (0.6 µg), and packaging plasmid as pAX2 (5.4 µg) (21). Cells were transfected using polyethyleneimine (Sigma-Aldrich) in Opti-MEM (Gibco) and serum-free Iscove’s modified Dulbecco’s medium (IMDM, Wisent Corp.). After 5 h of transfection, the culture medium was replaced with IMDM complete medium (IMDM supplemented with 10% vol/vol fetal bovine serum, 1% penicillin/streptomycin, 0.2% amphotericin B; Wisent Corp.). Supernatant containing lentivirus particles was harvested 72 h post-transfection and filtered through a 0.45-µm filter to remove cell debris. Virus aliquots were stored at −80°C until further use.

### DYRK1A depletion in HeLa B2 cell line

Depletion assays were done in six-well plates as previously described (16). Briefly, 4.0 × 10^5^ cells were seeded per well in IMDM complete medium. Prior to infection, shRNA lentiviral titers were determined using alamarBlue Cell Viability Reagent (Thermo Fisher Scientific) after puromycin selection. Cells were infected with shRNA lentivirus in complete medium containing 21.4-µM polybrene for 24 h. Following infection, fresh complete IMDM medium was added containing the selection agent, puromycin (4.24 µM), and incubated for 72 h. After selection, HIV-1 gene expression was induced by addition of Dox (4.5 µM) and was treated with dimethyl sulfoxide (DMSO) or harmine (10 µM) at the same time. Cells were harvested exactly 24 h following induction and compound or DMSO treatment and lysates processed for western analysis.

### Compound treatment assays

#### Screening assays

Screening of compounds (L41 [10 µM], TG003 [10 µM], KH-CB19 [30 µM], and harmine [10 µM]; Sigma-Aldrich) for their effect against HIV-1 was done in HeLa B2 cells. To evaluate the compounds’ (L41 [1 mM], TG003 [50 µM], KH-CB19 [50 µM], harmine [25 µM], 1H3 [250 nM], and 2E3 [250 nM]) effect on seasonal coronavirus HCoV-229E strain, Huh7 cells were used. Compounds were prepared by dissolving in dimethyl sulfoxide, aliquoted, and stored at −20°C. For screening against HIV-1, cells were seeded on a 12-well dish in IMDM complete medium and the following day induced with Dox and treated with DMSO or compounds at the same time. Cells were harvested 24 h after treatment, and lysates were examined for HIV-1 Env gp160, Gag, and Tat p16 and p14 protein levels by Western blotting. For screening against HCov-229E, Huh7 cells were seeded on a 96-well dish in DMEM containing 10% FBS (D10), and the following day, cells were infected with the virus at an multiplicity of infection (MOI) of 2 in serum-free DMEM (D0) for an hour, after which the virus was removed and cells were treated with the above compounds in DMEM supplemented with 2% FBS (D2). Sups were harvested 24 h post-infection (hpi) to quantify viral genomic RNA release in media by RT-qPCR assay.

#### Cytotoxicity assays of harmine

Cytotoxicity of harmine was assessed by using alamarBlue Cell Viability Reagent (Thermo Fisher Scientific) and expressed relative to cells treated with DMSO (1%). alamarBlue reagent was added to culture medium prior to harvest/fixation, cells were incubated at 37°C in a 5% CO_2_ humidified incubator for 2–6 h, and fluorescence reflecting cell metabolic rate was measured using Bio Tek Cytation 5 (Winooski, VT, USA). Fluorescence was monitored at an excitation wavelength of 530 nm and emission wavelength of 590 nm.

#### Dose-response assays

Harmine’s dose-response assays against HIV-1 were done in HeLa B2 as well as CEM-HIV cells. Briefly, HeLa B2 or CEM-HIV cells were plated on a 12-well dish and cells were induced with Dox (4.5 µM) or Dox + (4.5/2.56 µM), respectively, and treated with twofold serial dilutions of harmine using a 50-µM starting concentration. Cells were harvested 24 h post-treatment, and cell lysates were examined for changes in expression levels of HIV-1 Gag p55 and GagzipGFP protein levels by Western blotting. Dose-response assay of harmine against 229E was done in Huh7 cells. Following infection with the virus as described above, cells were treated with 100-µM starting concentration of the compound, which was serially diluted by twofold. Sups were harvested 24 hpi for RT-qPCR assay to measure viral RNA release in media.

#### Harmine’s effect on HIV-1 gene expression

One million CEM-HIV cells per well in a six-well dish were pelleted and resuspended in Rosewell Park Memorial Institute (RPMI) complete medium and induced with Dox + prostratin and treated with DMSO or harmine at the same time. Cells were harvested 24 h post-treatment for Western blotting of HIV-1 proteins and analysis of viral US, SS, and MS RNA levels.

#### Harmine treatment of primary cells

For the assays in primary CD4^+^ T cells, activated CD4^+^ T cells were spinoculated with HIV-1_89.6_ for 1 h followed by an additional 3-h incubation at 37°C. Virus was aliquoted onto 5 million cells per treatment condition, at 1 million cells per well of a 96-well flat bottom plate in 50 µL of R10 media with 10-ng/mL interleukin (IL)-2 (R&D Systems). The volume of virus added to each well of CD4^+^ T cells was determined based on the viral tittering of each stock that yielded saturated levels of infection, which typically ranged from ~25% to 50%, depending on the donor. After infection, the cells were pooled, washed once in R10 media to remove the virus, and then plated out in six-well plates at 5 million cells/well in 4 mL of R10 + 10-ng/mL IL-2 + inhibitor (1% DMSO or combined anti-retroviral drug Kaletra (lopinavir/ritonavir, 53 nM) + Epivir (lamivudine, 1.5 µM) or 10-µM harmine). After 3 days, the cells were harvested; 10% was used for flow cytometry analysis; 45% was used for Western blot analysis (lysed at 5 million cells per 100 µL of radioimmunoprecipitation assay (RIPA) buffer), and 45% was used for the RNA analysis (lysed in 300 µL of Aurum Total RNA Lysis Kit from Bio-Rad).

For macrophages, media were removed from 7-day differentiated macrophages, and fresh macrophage media (R10 containing 50-ng/mL granulocyte macrophage-colony stimulating factor (GM-CSF) and 50 ng/mL macrophage-colony stimulating factor (M-CSF)) were added back to each well. The volume of virus added to each well of macrophages was determined based on the viral tittering of each stock that yielded saturated levels of infection, typically ranging from ~10% to 50%, depending on the donor. The cells were incubated for 6 h at 37°C, followed by removal of the virus and addition of the original conditioned media diluted 1-in-2 with fresh macrophage media (19, 20). After 2 days of infection, the cells were pooled and set up for long-term cultures. Briefly, the macrophages were washed in PBS and lifted from the low attachment plates using Cell Dissociation Buffer (Thermo Fisher Scientific, Cat#13151014) for 10 min at 37°C, pooled and plated at 250,000 cells per well of a 24-well low attachment plate (Sigma-Aldrich, Cat#CLS3471) in 500 µL of macrophage media with either DMSO, Epivir/Kaletra, or harmine (10 µM). At day 4 post-treatment, half of the media was exchanged for new media containing fresh preparations of the inhibitors. On day 10 post-treatment, the macrophages were lifted from the plate and transferred to a 96-well V-bottom plate.

To measure SR kinase (CLK1, CLK2, CLK3, and SRPK1) and SR protein expression levels in mock infected primary CD4^+^ T cells, after 7 days of activation, the cells were treated with DMSO control or 10 µM of harmine for 72 h in R10 media and cell lysates harvested for western blots.

#### Effect of other monoamine oxidase A inhibitors on HIV-1 protein levels

HeLa B2 and CEM-HIV cells were used to examine the effect of other monoamine oxidase A inhibitors, harmane (22) (10 µM) and moclobemide (23) (30 µM), for their anti-HIV-1 activity together with harmine. Cells were plated on 12-well dish and induced and treated with compounds, after which the lysates were harvested to monitor HIV-1 protein levels by western blotting.

#### Effect of harmine on coronaviruses

To examine the effect of harmine on HCoV-229E replication, Huh7 cells were seeded on a six-well dish and infected with the virus at an MOI of 2 for 1 h, after which the virus inoculum was removed, and the cells were treated with harmine (25 µM). Media were harvested 24 hpi to measure extracellular viral RNA release by RT-qPCR. Additionally, cell lysates were harvested to monitor intracellular viral nucleocapsid (N) protein levels by Western blotting and accumulation of intracellular viral total and genomic RNA by RT-qPCR assays.

To study the effect of the compound on SARS-CoV-2 replication, cells were infected with virus at an MOI of 2 for an hour, then treated with harmine at different doses (25.0, 7.5, and 2.5 µM), and culture media were harvested at 24 hpi to measure SARS-CoV-2 RNA release by RT-qPCR. To determine changes in SARS-CoV-2 intracellular N protein levels and intracellular genomic and total RNA accumulation, cells were treated with DMSO or 25-µM harmine following infection and lysates were harvested 24 hpi for Western blotting and RNA analyses by RT-qPCR assay, respectively.

For viral outgrowth assay or time of incubation assay, Huh7 cells were seeded in a 96-well dish, and cells were infected with HCoV-229E as described above and treated with DMSO or 25-µM harmine. Culture media were then harvested at different time points (24, 48, and 72 hpi) to measure viral RNA release by RT-qPCR assay. TCID_50_ assay was then performed on media harvested after 72 h treated with DMSO or harmine. For TCID_50_, cells were seeded on a 96-well dish and infected with 10-fold serial dilutions of the viral sups in quadruplicate for an hour, after which the inoculum was washed off and cells were replenished with 150 µL of D2. After 5–7 days of incubation at 37°C, wells were examined for color change macroscopically and under the microscope for cytopathic effect (CPE). Wells showing CPE were marked, and TCID_50_/mL was calculated using the Spearman & Kärber calculator (24). For time of addition (TOA) studies, following infection, DMSO or harmine was added at various times (0, 12, 16 h) post-virus removal, and media were harvested at 24 h post infection to assay viral RNA levels by RT-qPCR.

To examine kinetics of HCoV-229E extracellular RNA release and intracellular genomic and total RNA and N protein accumulation, media and lysates from virus-infected cells were harvested at different time points post-compound addition. Briefly, Huh7 cells were infected with the virus as detailed above, and DMSO or harmine was added at 16 hpi, after which media and cell lysates were harvested at 0, 4.5, and 9.0 h post-compound addition. Media collected were processed for RT-qPCR to determine viral RNA release in media, and RNA was extracted (Bio-Rad Aurum Total RNA Mini Kit) from collected cell lysates and processed for RT-qPCR to determine accumulation of viral genomic and total RNAs. Cell lysates were also used to determine accumulation of N protein levels by Western blotting.

### Western blot analyses

For Western blotting, protein extracts were prepared by cell lysis in Radioimmunoprecipitation assay buffer (RIPA) buffer (50-mM Tris-HCl pH 7.5, 150-mM NaCl, 1% NP-40, 0.5% sodium deoxycholate, and 0.1% SDS) supplemented with Halt Phosphatase Inhibitor (Thermo Fisher Scientific). The extracts were fractionated on 10% TGX acrylamide stain-free gels (Bio-Rad) or 14% SDS-PAGE. Stain-free gels were directly imaged on ChemiDoc MP Imager (Bio-Rad) to measure total protein levels (served as loading control) prior to transfer to polyvinylidene difluoride (PVDF). Gels were directly imaged to measure GagzipGFP expression in CEM-HIV cells. Following imaging, proteins were transferred to PVDF (0.45 µM, Bio-Rad) using Trans-blot Turbo Transfer System (Bio-Rad). Immunoblots were blocked in 5% milk-TBS (Tris-buffered saline)-T (5% milk, 0.05% Tween-20, 1× TBS) or 5% bovine serum albumin (BSA)-TBS-T (5% BSA, 0.05% Tween-20, 1× TBS) for 1 h at room temperature (RT) prior to incubating in primary antibodies. After primary antibody incubations, blots were washed in 1× TBS-T and incubated in appropriate horse-radish peroxidase (HRP)-conjugated secondary antibody for an hour at RT. Following subsequent washes, blots were developed using Clarity Western ECL substrate (Bio-Rad) and imaged on ChemiDoc MP Imager (Bio-Rad). Band intensity was quantified relative to control in the experiment (DMSO or shControl) and normalized to the corresponding bands of the loading control (total protein for 10% gels and glyceraldehyde 3-phosphate dehydrogenase (GAPDH) for 14% gels) using ImageLab software (Bio-Rad). Details of all the primary antibodies used in this study are shown in Table S1.

### RNA analyses

#### Quantitation of HIV-1 mRNA levels

HIV-1 mRNA levels were assessed as previously detailed (16). The sequences of forward and reverse primers used for quantitation of HIV-1 unspliced (US), singly spliced (SS), and multiply spliced (MS) RNA levels and reference gene β-actin have been previously described (16). The quantitative PCR (qPCR) cycling conditions were as follows: activation at 95°C for 3 min followed by 40 cycles of denaturing at 95°C for 15 s, annealing at 55°C for 25 s, and extension at 68°C for 30 s. The melting curve protocol followed with 15 s at 95°C and then 15 s each at 0.2°C increments between 55°C and 95°C. Melting and standard curves were generated by the CFX Maestro Software (version 1.1, Bio-Rad).

#### Analysis of splice-site selection within HIV-1 MS RNA

Harmine’s effect on splice-site selection within HIV-1 MS RNA was analyzed as previously detailed (25).

#### Quantitation of HCoV-229E and SARS-CoV-2 RNA levels

For quantitation of viral RNA release in media (extracellular RNA), media were harvested and heat-inactivated immediately at 95°C for 5 min and directly used on Luna Universal One-Step RT-qPCR Kit (New England Biolabs, Ipswich, MA, USA) as per the manufacturer’s instructions. Each reaction was set up as follows: 5-µL Luna Universal One-Step Reaction Mix (2×), 0.5-µL Luna WarmStart RT Enzyme (20×), 0.2 µL of each 5′ and 3′ primers (10 µM), and 1 µL of media (template RNA) in a total reaction volume of 10 µL. The qPCR cycling conditions were as follows: reverse transcription at 55°C for 10 min and initial denaturation at 95°C for 1 min, followed by 40 cycles of denaturation at 95°C for 10 s and extension at 60°C for 30 s. The melting curve protocol followed with 15 s at 95°C and then 15 s each at 0.2°C increments between 60°C and 95°C. Melting and standard curves were generated by the CFX Maestro Software (version 1.1, Bio-Rad, Mississauga, ON, Canada). Sequences used to detect HCoV-229E and SARS-CoV-2 RNA in media have been previously described (5).

For quantitation of intracellular viral (HCoV-229E and SARS-CoV-2) genomic RNA and total RNA, cell lysates were harvested in Total RNA Lysis Buffer (Bio-Rad), and RNA samples were extracted using Aurum Total RNA Mini Kit (Bio-Rad #7326820EDU) following the manufacturer’s instructions with the addition of Turbo DNase (Ambion). Purified RNA (0.5 µg) was reverse transcribed using M-MLV reverse transcriptase (Invitrogen) as previously detailed (26). cDNA reactions (20 µL) were diluted to 100 µL and quantified for HCoV-229E genomic and total mRNA abundance by quantitative PCR using CFX384 Touch Real-Time PCR Detection System (Bio-Rad). Standard curve method was used for the quantitation of viral mRNA levels, normalized to the housekeeping gene, β-actin, and expressed relative to DMSO. Each reaction was set up as follows in qPCR 384-well plate: 5 µL of SsoAdvanced Universal SYBR Green Supermix (2×), 0.03 µL of each 5′ and 3′ primers (10 µM), and 1 µL of sup (template RNA) in a total reaction volume of 10 µL. The sequences of forward and reverse primers used for quantitation of HCoV-229E genomic RNA are 5′ TGGGACTATCCTAAGTGGAT 3′ and 5′ GTACCACCAGGTTTAAAATAAA 3′, and total RNA are 5′ TATTATCTTGGCACAGGACC 3′ and 5′ TGAAGGATTCCGAGATTGAG 3′. For quantitation of SARS-CoV2 RNAs, primers were targeted to either the Orf1ab region (genomic, 5′ CCCTGTGGGTTTTACACTTAA 3′ and 5′ ACGATTGTGCATCAGCTGA 3′) or N coding region (total RNA, 5′ CCTCTTCTCGTTCCTCATCA 3′ and 5′ CCTGGTCCCCAAAATTTCCT 3′). The sequences of the forward and reverse primer set for the reference gene β-actin are 5′ AGCTCATTGTAGAAGGTGTGG 3′ and 5′ GGCATGGGTCAGAAGGATTC 3′. The qPCR cycling conditions and the melting curve protocol were the same as detailed for sups. Standard curve method was used for the quantitation of viral genomic and total RNA levels, normalized to the housekeeping gene, β-actin, and expressed relative to DMSO.

### Flow cytometry analysis

For flow cytometry analysis of primary cells, macrophage samples were Fc blocked with Human TruStain FcX (BioLegend, Cat#422302) as per the manufacturer’s instructions. Once the Fc blocking was completed, macrophages and CD4^+^ T cells were surface stained with anti-CD14-Pacific Blue (BioLegend, Cat#301828; macrophages only), anti-CD3-Pacific Blue (BioLegend, Cat#300330; CD4+ T cells only), anti-CD4-APC (BioLegend, Cat#317416), and LIVE/DEAD Fixable Blue (Thermo Fisher Scientific, Cat#L34962), fixed and permeabilized using BD CytoFix/CytoPerm Fixation/Permeabilization Kit (BD Biosciences, Cat#554714), and stained for the HIV Gag protein using anti-Gag p24-FITC (Beckman Coulter, Brea, CA, USA; Cat#6604665). Flow cytometric data were acquired using a FACSCanto instrument with FACSDiva software (BD Biosciences). All data were analyzed using FlowJo (version 10.6.0 software; FlowJo, LLC, Ashland, OR, USA). Uninfected cell samples were used to draw the infected cell gate. Infected cells were considered LIVE/DEAD^Neg^, CD14^Int/Pos^CD4^−^p24^+^ (macrophages), or CD3^Pos^CD4^+^p24^+^ (CD4^+^ T cells). Frequencies of this population were compared between DMSO, Epivir + Kaletra, and harmine-treated samples. Within the infected population, the Gag mean fluorescence intensity (MFI) was also compared in infected macrophages.

### Immunofluorescence (IF) and fluorescent *in situ* hybridization (FISH)

Huh7 cells seeded on coverslips on 12-well dish were infected with HCoV-229E (MOI of 2) for an hour, after which the virus inoculum was washed off and cells treated with DMSO or harmine at 16 hpi. Cells were fixed with 4% paraformaldehyde in PBS 4.5 and 9.0 h post-compound treatment and processed for immunofluorescence or fluorescent *in situ* hybridization. For IF, fixed cells were permeabilized in 0.1% Triton X-100 in 1× PBS, washed twice in PBS, incubated in blocking buffer (0.5% blocking reagent [Roche, Cat #1096176] prepared in 100-mM M Tris-HCL, pH 7.5, 150-mM NaCl) for an hour at RT, and incubated in mouse ⍺-double-stranded RNA (dsRNA) monoclonal antibody (1:200 dilution in 1× PBS [Jena BioScience, #RNT-SCI-10010200]) and sheep ⍺229E nucleocapsid antibody (University of Dundee, Dundee, UK, Cat #DA115 [1:400 dilution in 1× PBS]) for an hour at RT. Following washing to remove unbound antibodies, coverslips were incubated in secondary antibodies (Alexa Fluor 488-conjugated AffiniPure Donkey ⍺-mouse IgG and Cy3 AffiniPure Donkey ⍺-sheep IgG [Jackson ImmunoResearch Laboratories Inc.]) for an hour, after which they were washed, stained for 4',6-diamidino-2-phenylindole (DAPI), and mounted on glass slides using ProLong Gold Antifade Mountant (ThermoFisher Scientific, #P36930). For FISH, fixed cells were dehydrated in 70% ethanol, then rehydrated in wash buffer (10% formamide, 2× SSPE [made by diluting 1 L of 20× sSSPE buffer that contains 175.3-g NaCl, 27.6 g of NaH_2_PO_4_·H_2_O, 7.4-g EDTA, pH adjusted to 7.4 with NaOH]). Hybridization was performed using HCoV-229E genomic or total RNA oligonucleotides spanning the regions of the virus as detailed. Hybridization was performed using HCoV-229E genomic or total RNA oligonucleotides spanning the regions of the virus as detailed in Table S2. Following washing with the wash buffer to remove unbound probe, nuclei were DAPI stained, and slides were mounted as detailed above. Images were acquired on Zeiss microscope with AxioCamICc 5 camera at 40× oil immersion using Zen software.

### RNA sequencing (RNA-Seq)

#### Sample preparation for RNA-Seq

Mock-infected CD4^+^ T cells treated with DMSO, harmine (10 µM) or 1H3 (200 nM) for 3 days (two donors per treatment condition) were used for sample preparation for RNA-Seq. Total RNA was extracted from treated samples using Aurum Total RNA Mini Kit (Bio-Rad #7326820EDU) following the manufacturer’s instructions with the addition of Turbo DNase (Ambion). RNA integrity was verified using an Agilent Technologies 2100 Bioanalyzer (RNA integrity number value ≥ 8) at Donnelley Sequencing Center (DSC, University of Toronto, Toronto, ON, Canada). Libraries were generated from 500 ng of total RNA using NEBNext Ultra II Directional RNA Library Prep Kit for Illumina (New England Biolabs, #E7490S). Polyadenylated mRNA enrichment was performed using the NEBNext Poly(A) mRNA Magnetic Isolation Module followed by slight modification on RNA fragmentation time (10 min at 94°C) for an insert size of 350 bp (to span exon-exon junction). The RNA fragments were then copied into cDNA using NEBNext First Strand Synthesis and Second Strand Synthesis modules, and the double-stranded cDNA was purified using SPRIselect beads. This was followed by end-prep of cDNA library and adapter ligation using NEBNext Ligation Enhancer and NEBNext Ultra II Ligation Master Mix. Adapter ligated DNA was then PCR enriched using NEBNext Ultra II Q5 Master Mix and NEBNext Multiplex Oligos for Illumina (Unique Dual Index UMI Adaptors DNA Set 1) followed by purification of PCR enriched reaction using SPRIselect beads. The quality of the library was assessed on an Agilent Bioanalyzer DNA 1000 Chip, normalized and pooled for cluster generation.

#### Sequencing and analysis of RNA-Seq data

cDNA libraries were sequenced on Illumina NovaSeq 6000 (Illumina, San Diego, CA, USA), SP 300 cycles (paired-end, 350 bp) at DSC, University of Toronto, Toronto, ON, Canada. The RNA-Seq data analyses were performed by the Université de Sherbrooke RNomics Platform (https://rnomics.med.usherbrooke.ca). RNA-Seq data obtained from DSC were demultiplexed and stored in FASTQ format. Sequence read data were preprocessed using Trimmomatic (27) to remove adapter read-through and trailing low-quality bases. FastQC was used to check the quality of the reads after trimming. The human genome GRCh38.p12 and annotation from ENSEMBL (Homo sapiens version 98) were used to create the transcriptome annotation. The reads were aligned, and transcripts were quantified using Kallisto version 0.44.0 software (28). Gene counts and gene transcript per million (TPM) were obtained by summing the corresponding value of each transcript of a gene. The differential gene expression (DGE) analysis was performed on the IRIS-EDA webserver using the DESeq2 package. The identified genes with absolute log_2_ fold change (|LFC|) of ≥ 2 and Benjamini-Hochberg adjusted *P* values of ≤ 0.05 were considered as differentially expressed from the DMSO control.

For changes in alternative splicing events (ASEs), associated gene isoforms were quantified in TPM using RNA-Seq by Expectation-Maximization (RSEM) for each sequenced sample (29). A maximum of two mismatches in the seed (25 bases) were allowed (default parameter). All candidate ASEs were automatically annotated from the RefSeq curated transcriptome database and quantified using the percent-spliced in (PSI) metric based on the abundance of long (L) and short (S) isoforms in the ratio: L/(L + S). The ASE list was filtered to keep only the events whose average S + L isoform expression was at least 2 TPM. To ensure higher stringency, the ASEs were further filtered with an adjusted *t*-test *P* value of ≤ 0.05. From these events, only those with changes in PSI value of greater than 10% were considered as a significant alteration.

Analysis of alternative polyadenylation from the RNA-Seq data was carried out using Quantification of Alternative Polyadenylation (QAPA) software, as previously described (30). The source code and the QAPA-annotated 3′ untranslated region (UTR) libraries for human (hg38) are available on GitHub (https://github.com/morrislab/qapa). The estimates of each isoform 3′ UTR expression level was obtained by mapping Fastq files to 3′ UTR libraries using an external transcript quantification program, Salmon (31). Only 3′ UTRs that were expressed at an average of at least 2 TPM in either of the data set and the 3′ UTR in protein coding genes were considered for further analyses. To quantify the relative usage of 3′ UTR isoforms, the relative expression of a 3′ UTR over the total expression level of all 3′ UTRs in a gene was calculated as poly(A) usage (PAU) metric (30). Since APA is often regulated through the differential usage of proximal poly(A) sites (32 – 34), we focused on the PAU of the proximal 3′ UTR (PPAU). Thus, a higher PPAU value indicates preference of proximal poly(A) site over the distal site (30). To quantify changes in APA in compound treated vs DMSO control samples, we calculated change in PPAU, i.e., ∆PPAU, by subtracting the PPAU of DMSO treatment from the PPAU of compound treatment. Genes with absolute ∆PPAU values of greater than or equal to 20% and *t*-test *P*-value of ≤ 0.05 were considered to have a significant differential usage of poly(A) site. Genes with ∆PPAU > 20% were deemed to have shortened 3’UTR, while genes with ∆PPAU < −20% were deemed to have lengthened 3′ UTR, and others (−20% < ∆PPAU < 20%) were considered as no change in 3′ UTR length. The PPAU comparison was restricted to genes with exactly two poly(A) sites, as previously described (30).

#### DGE validation by RT-qPCR

To validate the DGE data from RNA-Seq, some of the most significantly altered genes upon harmine or 1H3 treatment were quantified by RT-qPCR. Total RNA was extracted from DMSO, harmine or 1H3-treated samples as described in “*Sample preparation for RNA sequencing*” section. cDNA was synthesized from 0.5 µg of total RNA per sample (across two to four donors) using GoScript Reverse Transcription System (Promega) following manufacturer’s instructions. The cDNAs were diluted to a final volume of 20 µL and proceeded to qPCR. For qPCR, gene-specific primers were designed through https://pga.mgh.harvard.edu/primerbank/ or https://www.primer3plus.com/ websites, for coding sequences of the target genes. List of all the primers used to validate RNA-Seq data are listed in Table S3. qPCR reactions were set up as follows: 5 µL of SsoAdvanced Universal SYBR Green Supermix (2X) (Bio-Rad), 0.25 µL of each 5′ and 3′ primers (10 µM), and 1 µL of template cDNA in a total reaction volume of 10 µL. The qPCR cycling conditions were: initial denaturation at 95°C for 3 min, followed by 40 cycles of denaturation at 95°C for 30 s, annealing at 55°C for 30 s, and extension at 72°C for 30 s. The melting curve protocol followed with 15 s at 95°C and then 15 s each at 0.2°C increments between 55°C and 95°C. Melting and standard curves were generated by the CFX Maestro Software (version 1.1, Bio-Rad).

#### ASE validation by RT-PCR

For validation of ASEs observed in the RNA-Seq data, RT-PCR was exploited. The single exon skipping (SES) events with the most significant alteration in splicing upon harmine or 1H3 treatment were selected for validation. We then extracted full exonic and intronic sequences (alternative exon and flanking introns and exons) surrounding the single exon-skipping events from the UCSC Genome Browser. The forward and reverse primers were positioned on the upstream exon and downstream exon, respectively, surrounding the alternative exon. The primers for validation of ASEs have been listed in Table S3. For RT-PCR, cDNA templates were synthesized as detailed above. Each PCR reaction was set up as follows: 10 µL of SsoAdvanced Universal SYBR Green Supermix (2X) (Bio-Rad), 0.25 µL of each 5′ and 3′ primers (10 µM), and 1 µL of template cDNA in a total reaction volume of 20 µL. The PCR cycling conditions were as follows: initial denaturation at 95°C for 3 min, followed by 30 cycles of denaturation at 95°C for 30 s, annealing at 55°C for 60 s, and extension at 72°C for 60 s, with a final extension at 72°C for 5 min and an infinite hold at 4°C. Following PCR, the amplicons were run on 7–8% polyacrylamide (PAA) gels at 200 V for 1.5–2.5 h. Gels were soaked in distilled water containing 0.5 µg/mL of ethidium bromide and imaged in Chemidoc MP Imager (Bio-Rad).

## RESULTS

### Harmine inhibits HIV-1 protein expression

**Fig 1:**
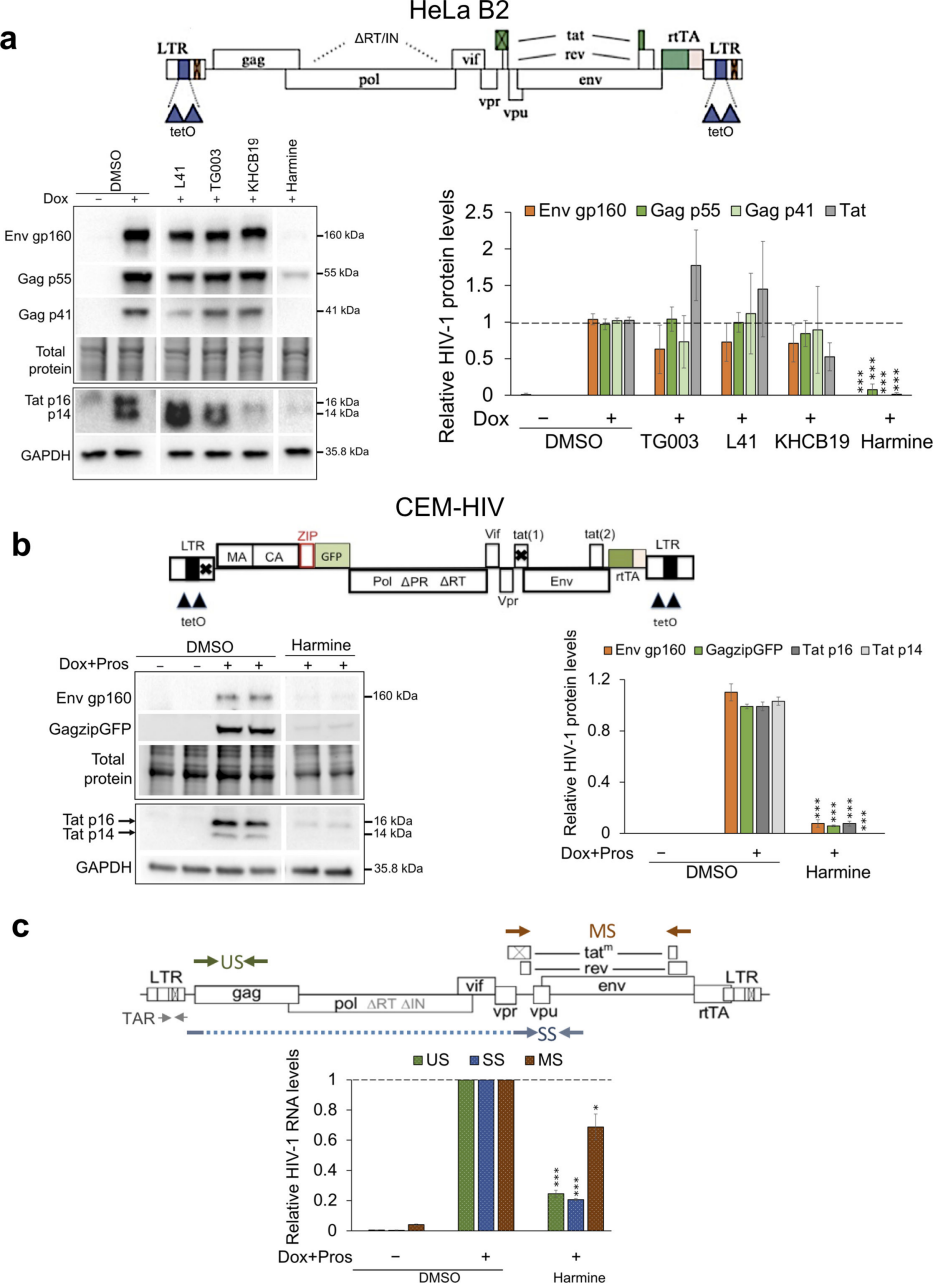
Evaluating SR kinase inhibitors for their anti-HIV-1 activity identified harmine as a potent hit. (**a**) Compounds L41 (10 µM), TG003 (10 µM), KH-CB19 (30 µM), and harmine (10 µM) were assessed in HeLa B2 cells for their effects on expression levels of HIV-1 Env, Gag, and Tat protein levels by Western blotting. At the top is the proviral construct present in the HeLa B2 cell line. On the left are representative Western blots and, on the right, the quantitation of the blots across six independent experiments. Dotted vertical lines on the blots represent cropping of lanes on the same representative blots to show harmine-treated lanes adjacent to all the SR kinase inhibitors evaluated. (**b**) Harmine’s effect on the expression levels of HIV-1 Env, GagzipGFP, and Tat protein levels in CEM-HIV cells as demonstrated by Western blotting. At the top is the proviral construct present in the CEM-HIV cell line. On the left are the representative blots, and on the right is the quantitation of the blots across three independent experiments performed in duplicates. In both panels** a** and **b, **thin tooling line between the blots represent splicing of the same representative blots exclusively for labeling purposes, and for both figures, band intensities were quantified relative to induced DMSO control and were normalized to either total protein load for Env and Gag blots or GAPDH for Tat blots using Bio-Rad ImageLab software. (**c**) Schematic of HIV-1 provirus indicating the positions of the primers used to detect viral RNAs and quantitation of HIV-1 unspliced (US), singly spliced (SS) and multiply spliced (MS) RNA levels by RT-qPCR in CEM-HIV cells treated with DMSO or harmine. Viral mRNA levels were normalized to β-actin, and the mean mRNA levels were expressed relative to DMSO control across three independent experiments performed in duplicates. Data are indicated as mean ± SD. *P ≤ 0.05, ***P ≤ 0.001.

In light of the dependence of HIV-1 on alternative RNA splicing for expression of its genome (2), and the role of SR proteins in regulating splice-site selection (35), we evaluated several SR protein kinase inhibitors (leucettine 41, TG003, KH-CB19, and harmine) (18) for their effect on HIV-1 gene expression using HeLa rtTA HIV∆mls (HeLa B2), a cell line containing a doxycycline (Dox)-inducible HIV-1 provirus (26). All drugs were tested at a final concentration of 10 µM (except KH-CB19 which was tested at 30 µM), the concentration shown to affect SR kinase function and/or SR protein phosphorylation (17, 36 – 40). Screening identified harmine as a potent hit, reducing HIV-1 Env gp160, Gag p55 and p41, and Tat p16 and p14 protein levels by more than 95% compared to the DMSO control (Fig. 1a). In contrast, the other inhibitors tested had minimal effect. Dose-response and cytotoxicity assays with harmine revealed dose-dependent inhibition of HIV-1 Gag p55 expression with an IC_50_ of 4.7 µM and, at the dose (10 µM) used in the cell system, there was minimal impact on cell viability (CC_50_ >50 µM) (Fig. S1a). Subsequent tests in a T cell line containing a Dox inducible HIV-1 provirus, CEM-HIV cells (16), showed a similar dose-dependent inhibition of HIV-1 GazipGFP levels with a CC_50_ >50 µM and an IC_50_ of 1 µM (Fig. S1b). Reduction of HIV-1 Env gp160, GagzipGFP, and Tat p16 and p14 protein levels (Fig. 1b) was similar to that seen in the HeLa cell line.

### Harmine alters HIV-1 RNA accumulation

To understand the basis for the reduction in HIV-1 protein levels upon harmine treatment, we examined whether it was correlated with changes in the abundance of HIV-1 unspliced, singly-spliced, and multiply-spliced (MS) RNAs by RT-qPCR assay. Total RNA was extracted from CEM-HIV cells treated with DMSO or harmine (10 µM), and RT-qPCR assays were performed for all the three classes of viral RNAs. The positions of the primers in the proviral construct are shown in Fig. 1c. Harmine treatment reduced accumulation of US and SS viral RNAs consistent with the loss of HIV-1 Gag, Env, and Tat p14 protein expression. However, a significant accumulation of MS transcripts was observed despite loss of Tat p16 encoded by MS RNA (Fig. 1c). To determine if the compound alters the level of splice variants within HIV-1 MS RNA species, RT-PCR was performed to analyze the HIV-1 MS RNA splicing pattern using forward and reverse primers that amplify the MS RNA spliced isoforms. No significant changes in splice-site usage were observed upon harmine treatment that would contribute to the alterations in the viral Tat p16 expression observed (Fig. S1c). Thus, harmine caused minimal alterations in the utilization of HIV-1 splice sites in the MS RNAs, implying that its impact lies in modifying the overall splicing of the primary HIV-1 transcripts rather than altering the use of specific splice sites.

### Effect of harmine on HIV-1 replication in primary CD4^+^ T cells/macrophages

**Fig 2:**
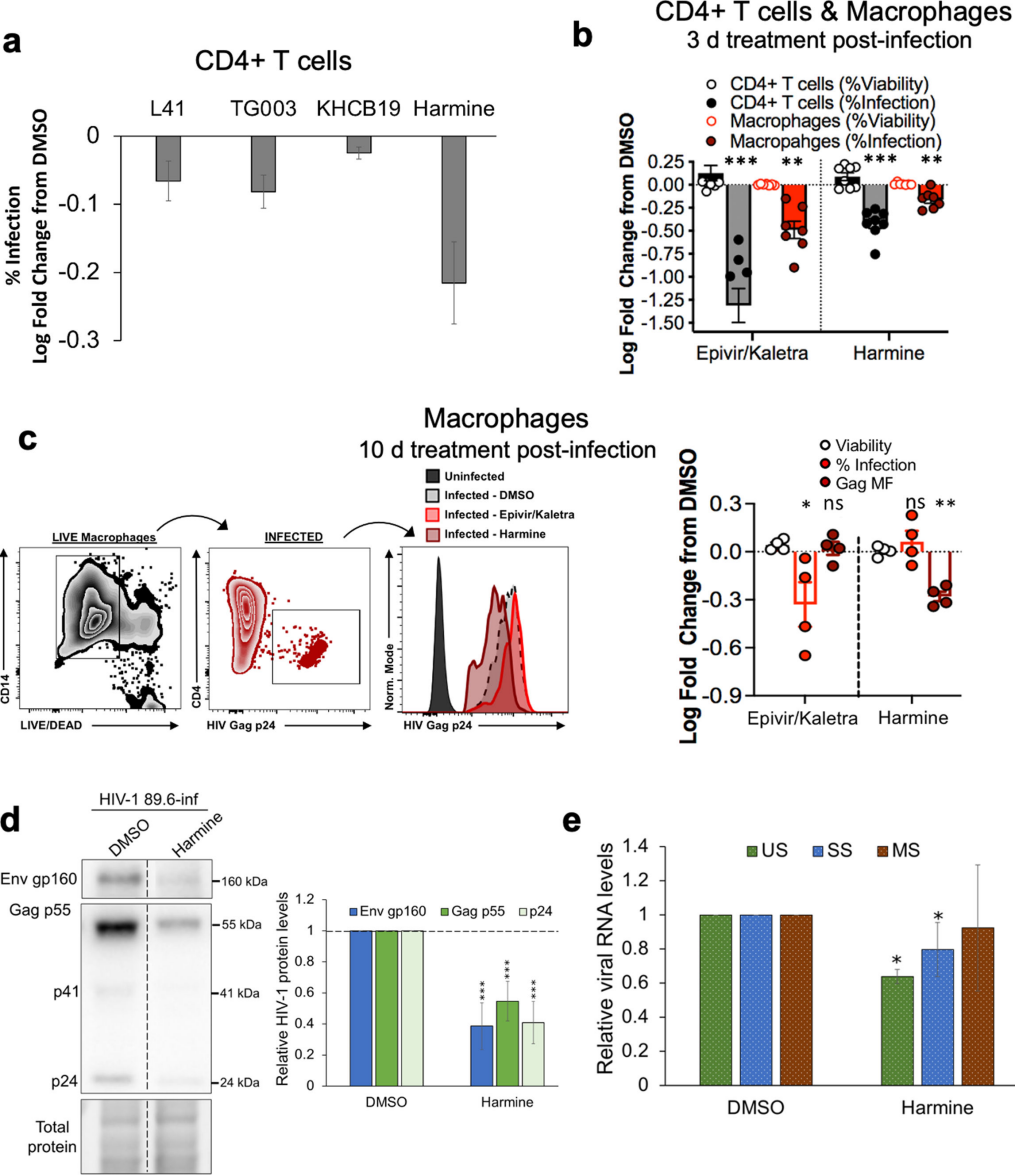
Effect of harmine on HIV-1 in CD4^+^ T cells and macrophages. (**a**) Evaluation of the effect of SR kinase inhibitors, L41 (10 µM), TG003 (10 µM), KH-CB19 (30 µM), or harmine (10 µM), over a period of 3 days, in healthy donor CD4^+^ T cells infected with wild-type HIV-1_89.6_. (**b**) Compound treatment assays in CD4^+^ T cells or monocyte-derived macrophages infected with HIV-1_89.6_ and treated with harmine (10 µM) or Epivir/Kaletra over a period of 3 days, after which cells were fixed, and % viability and % infection (Gag-positive cells) were assessed by flow cytometry. The fixable viability dye, LIVE/DEAD stain (Thermo Fisher Scientific), was used to assess cell health. (**c**) Compound treatment assays repeated in macrophages over a period of 10 days, after which cells were fixed, and % viability, % infection, and Gag mean fluorescence intensity were assessed by flow cytometry. On the left are the representative flow plots of infected macrophages stained for intracellular HIV-1 Gag (P24**4**), indicated cell surface markers (macrophages, anti-CD14, and CD4), the gating strategy used, and on the right are the summary data expressed relative to DMSO-treated samples. (a through c) The experiments were performed across four to six donors. (**d and e**) Healthy CD4^+^ T cells from donors were infected with HIV-1_89.6_ and treated with DMSO or harmine (10 µM) for 3 days. Cells were harvested, and the effect of individual treatments on HIV-1 (**d**) proteins or (**e**) RNA accumulation was assessed by Western blotting and RT-qPCR, respectively. Shown are the summary of the results from six independent experiments with six donors. Data are indicated as mean ± SD. *P ≤ 0.05, **P ≤ 0.01, and ***P ≤ 0.001. Dotted vertical lines on the blots represent cropping of lanes on the same representative blots to show harmine-treated lanes adjacent to DMSO-treated lanes. ns, not significant.

To assess whether the effects of harmine observed in cell lines could be translated into primary cells, we screened the same panel of SR kinase inhibitors for their effect on the growth of wild-type HIV-1_89.6_ (an R5 × 4 dual-tropic primary isolate of HIV-1) in CD4^+^ T cells obtained from healthy (HIV-uninfected) human donors over 3 days. As observed in studies using cell lines, harmine addition resulted in the greatest reduction in virus replication among the SR kinase inhibitors tested as measured by intracellular Gag staining (Fig. 2a). DMSO, harmine, or combined anti-retroviral Epivir/Kaletra (lamivudine and lopinavir/ritonavir) were subsequently assessed in donor CD4^+^ T cells as well as macrophages infected with HIV-1_89.6_ over a period of 3 days. As shown in Fig. 2b, treatment with either harmine or Epivir/Kaletra reduced the frequency of HIV-1 infection in CD4^+^ T cells reflecting an inhibition of the virus expansion within the culture. However, harmine addition had no impact on the frequency of infected macrophages. We further exploited the ability of the macrophages to survive the cytopathic effect of infection and thus assessed the compound effects over long term (10 days) in these cells (41, 42). Two days post-infection, macrophages were treated with DMSO, Epivir/Kaletra, or harmine for 10 days, followed by flow cytometry-based analysis of percent viability, percent infection, and levels of intracellular Gag p24 expression. Harmine treatment of macrophages did not reduce percent infection compared to DMSO but did reduce the Gag p24 mean fluorescence intensity of infected macrophages, unlike Epivir/Kaletra treatment that did not reduce Gag MFI despite reduced infection (Fig. 2c). The decrease in Gag MFI of infected macrophages upon harmine addition indicates that the compound reduced the amount of viral protein expression in infected cells. Anti-viral effects were observed without any significant reduction in cell viability for the treatments evaluated (Fig. 2b and c). Consistent with the effects measured by flow cytometry, harmine addition to CD4^+^ T cells infected with HIV-1_89.6_ reduced HIV-1 Env gp160 and Gag protein levels as measured by Western blot (Fig. 2d). Similar to the effect observed in the cell line, the compound reduced US and SS RNA abundance (Fig. 2e) with more limited changes in MS RNA levels upon addition to CD4^+^ T cells.

### Harmine’s anti-HIV-1 activity is independent of DYRK1A and MAO A

**Fig 3:**
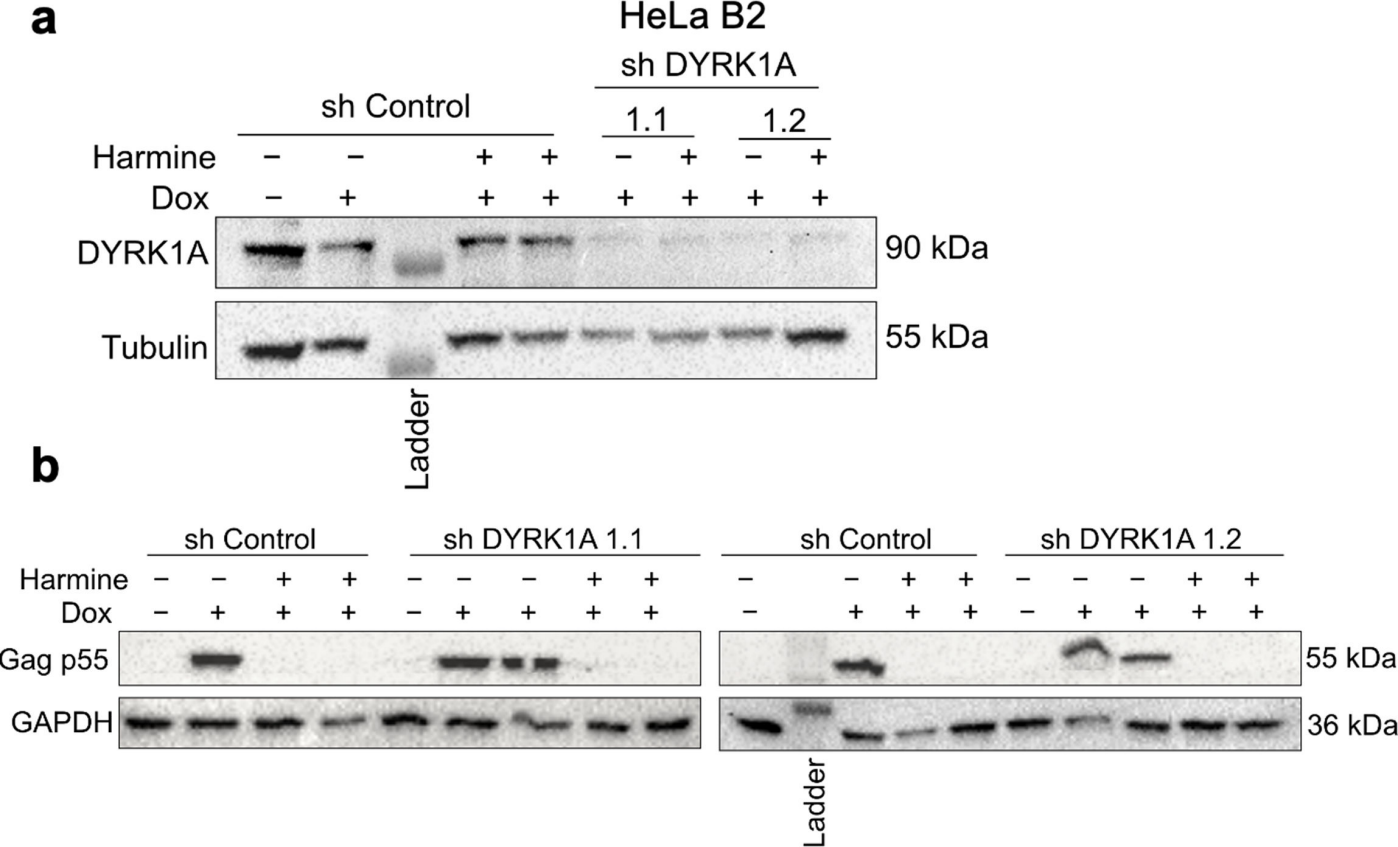
Effect of DYRK1A depletion on harmine’s anti-HIV-1 activity. HeLa B2 cells were infected with shRNA lentiviruses targeting DYRK1A (shDRYK1A 1.1 and shDYRK1A 1.2) and transduced cells selected with puromycin for 72 h. Following puromycin selection, HIV-1 gene expression was induced with Dox (4.5 µM), and cells were treated with DMSO or harmine (10 µM) and harvested for Western blots after 24 h. (**a**) Representative Western blots confirming DYRK1A depletion. (**b**) Representative blots showing Gag p55 expression levels. Antibodies against tubulin or GAPDH were used as loading control.

Harmine is a known inhibitor of the SR kinase, DYRK1A, both *in vitr*o and in cultured cells (43 – 45). To determine if the anti-HIV-1 activity of harmine is due to inhibition of DYRK1A, we examined the effect of DYRK1A depletion on HIV-1 gene expression with or without harmine treatment. Reduction of DYRK1A expression by ~90% (Fig. 3a) in the absence of harmine had no effect on HIV-1 Gag expression (Fig. 3b). Furthermore, harmine retained its anti-HIV-1 activity even in DYRK1A depleted cells (as indicated by the reduction in viral Gag p55 protein levels, Fig. 3b), suggesting that harmine’s anti-HIV-1 activity is independent of DYRK1A.

In addition to DYRK1A, harmine also inhibits monoamine oxidase A (43 – 45). To determine if the compound affects HIV-1 through inhibition of MAO A, we tested two other MAO A inhibitors, harmane (22) and moclobemide (23), for their ability to affect HIV-1 gene expression in two different cell lines: HeLa B2 and CEM-HIV cells. In contrast to harmine, neither harmane nor moclobemide significantly reduced HIV-1 gene expression in either of these cell systems (Fig. S2a and b), indicating that harmine’s anti-HIV activity is mediated by affecting other pathways.

### Harmine increases CLK1, reduces CLK2 kinase levels, and alters abundance of select SR proteins

**Fig 4:**
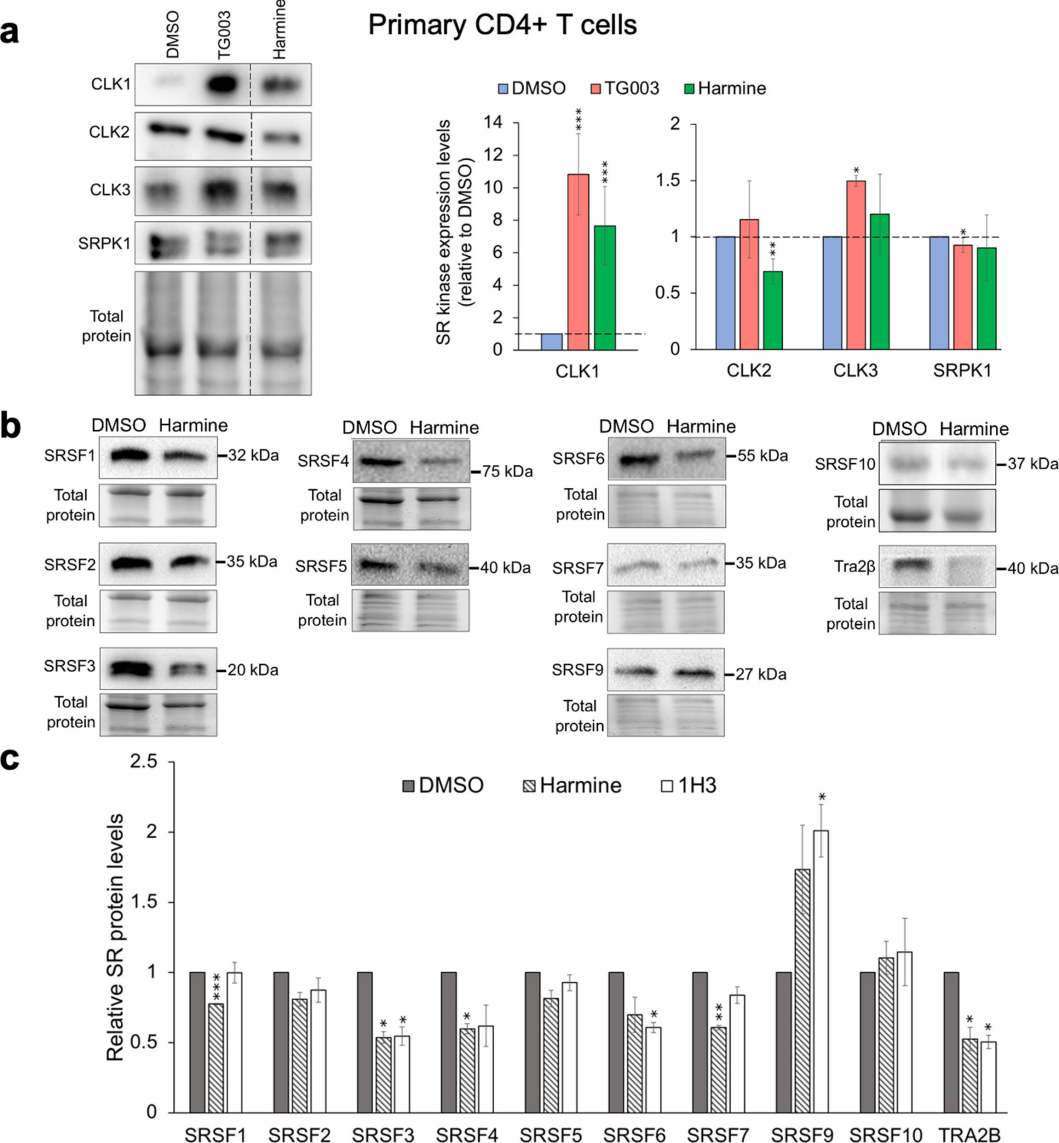
Harmine alters CLK1 and CLK2 expression levels and selectively changes abundance of SR proteins. (**a**) CD4^+^ T cells from healthy donors were infected with HIV-1_89.6_ and treated with DMSO, TG003 (10 µM), or harmine (10 µM) for 3 days. Cells were harvested, and the effect of individual treatments on SR kinase levels (CLK1, CLK2, CLK3, or SRPK1) was determined by Western blotting. Shown on the left are the representative Western blots, and on the right is the quantitation of the blots for *n* = 6 individual donor samples. Dotted vertical lines on the blots represent cropping of lanes on the same representative blots to show harmine-treated lanes adjacent to DMSO-treated lanes. (**b**) Representative blots showing expression levels of indicated SR proteins in T cells treated with DMSO or harmine for 3 days. The gel images for total protein for SRSF3 and SRSF4 are reused because they are part of the same internally controlled experiment. (**c**) Quantitation of SR protein levels from the Western blots of three independent donor samples treated with harmine (10 µM) or 1H3 (300 nM). Band intensities were quantified relative to DMSO control and were normalized to total protein using Bio-Rad ImageLab software. Data are indicated as mean ± SD. *P ≤ 0.05, **P ≤ 0.01, ***P ≤ 0.001.

A previous kinome scan of harmine (10 µM) against 468 human kinases determined that, in addition to DYRK1a, the compound also inhibited multiple CMGC kinases, including the CLK family of SR kinases (46). To determine if harmine has any impact on the expression levels of these SR kinases, we examined its effect on CLK1, CLK2, CLK3, and SRPK1 levels. Treatment of primary CD4^+^ T cell with harmine or a known CLK1 inhibitor, TG003 (36), increased CLK1 expression levels by ~8 to 10-fold. However, unlike TG003, harmine addition also reduced CLK2 expression levels by ~30% (Fig. 4a). These data suggest that harmine‘s anti-HIV-1 activity might be due to changes in the relative abundance of CLK1 and CLK2 protein levels.

To determine if harmine-induced changes in SR kinase expression affected SR protein abundance, we monitored expression of multiple SR proteins (SRSF1–7, 9, 10, and Tra2ß) following treatment of primary, activated CD4^+^ T cells with DMSO, harmine, or a recently described HIV-1 inhibitor, 1H3 (GW801372X) that also works by affecting SR kinase function/expression (16). As shown in Fig. 4b and c, harmine treatment of primary cells altered expression of several SR proteins in a pattern similar to that seen upon addition of 1H3: a reduction of SRSF3, SRSF4, and SR-related protein Tra2ß with slightly increased SFSR9 expression. Harmine also reduced SRSF1 and 7 levels, but expression of these host factors was not affected by 1H3 addition (16).

### Harmine inhibits coronavirus replication

**Fig 5:**
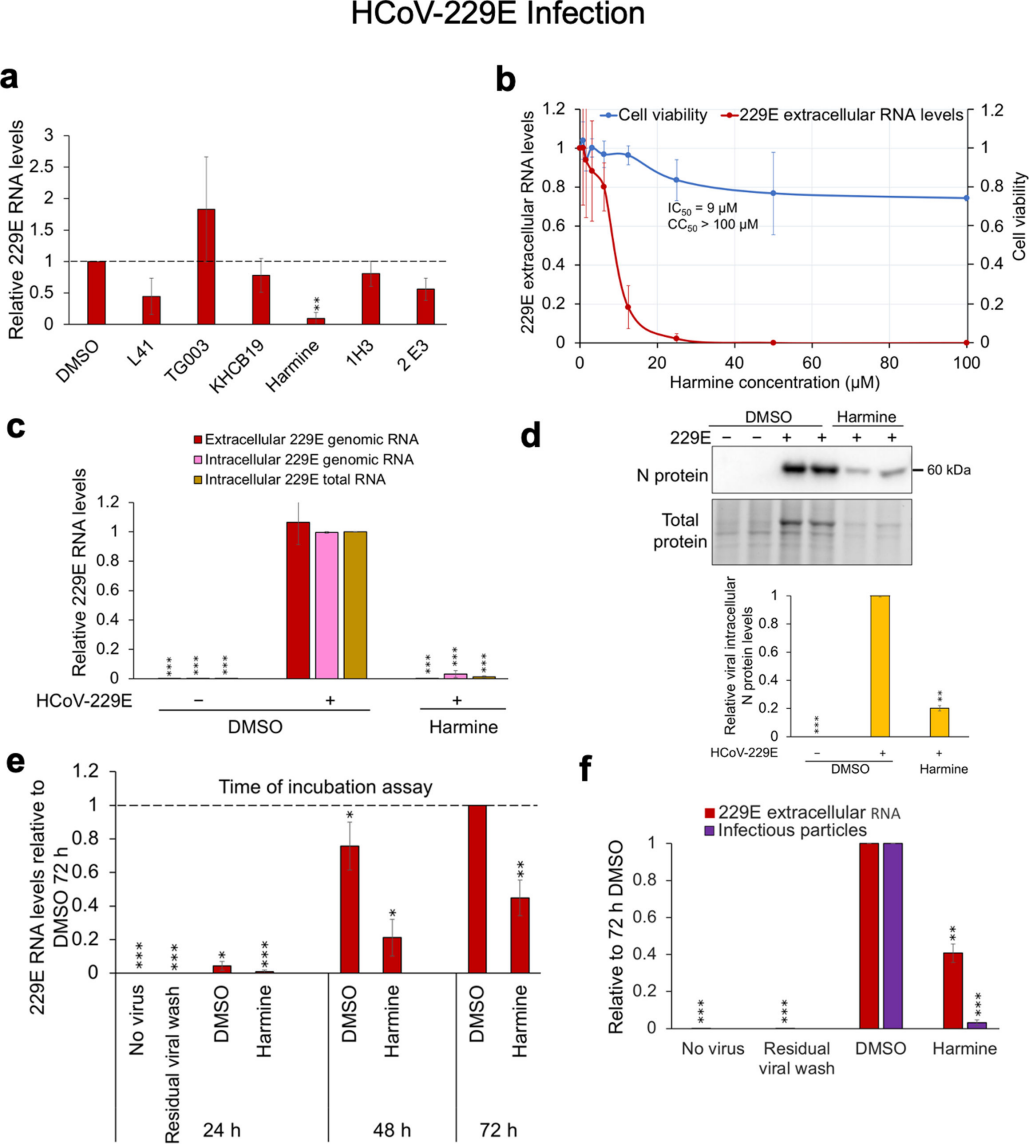
Harmine is an inhibitor of HCoV-229E replication. (**a**) Compounds L41 (50 µM), TG003 (10 µM), KH-CB19 (50 µM), harmine (25 µM), 1H3 (250 nM), and 2E3 (250 nM) were screened for their ability to inhibit HCoV-229E (MOI of 2) replication in Huh7 cells. Viral sups were harvested 24 hpi to measure extracellular RNA release in media by RT-qPCR assay. (**b–d**) Huh7 cells were infected with HCoV-229E virus at an MOI of 2 and were treated with DMSO or harmine for 24 h. Sups were harvested at 24 hpi for extracellular viral RNA analysis by RT-qPCR, and lysates were harvested for intracellular RNA and protein analyses. (**b**) Dose-response curve showing dose-dependent inhibition of viral extracellular RNA levels with increasing harmine concentration. Cell viability was assessed by alamarBlue. (**c**) Effect of harmine (25 µM) on extracellular viral RNA release and intracellular viral genomic and total RNA accumulation as measured by RT-qPCR. (**d**) Representative Western blots showing HCoV-229E nucleocapsid (N) protein levels in DMSO or harmine (25 µM)-treated samples. On the bottom of the blots is the quantitation of *n* = 3 independent experiments performed in duplicates. Band intensity was quantified relative to DMSO control and was normalized to total protein using Bio-Rad ImageLab software. (**e**) Viral outgrowth/time of incubation assay in which the DMSO- or harmine-treated sups (media) were harvested at 24, 48, and 72 hpi, and viral RNA release measured by RT-qPCR assay. (**f**) Measurement of infectious particles released over 72 h of DMSO or harmine-treated (25 µM) samples by TCID50 assay. Data are indicated as mean ± SD. *P ≤ 0.05, **P ≤ 0.01, ***P ≤ 0.001.

Given the demonstrated role of some SR kinases in the regulation of coronavirus replication (14, 47) and an intense interest in developing novel therapies for the current COVID-19 pandemic, we tested the same panel of SR kinase inhibitors for their effect on replication of a seasonal coronavirus strain, HCoV-229E, at doses that did not reduce cell viability. Compound screening in Huh7 cells identified harmine as the most potent inhibitor of HCoV-229E replication at the doses used, reducing viral RNA release into media (a measure of virion release in media) by more than 95% (Fig. 5a). Subsequent dose-response assays revealed a dose-dependent inhibition of viral RNA release in media with an IC_50_ of 9 µM and CC_50_ of >100 µM over 2 days of compound treatment (Fig. 5b). Harmine treatment (25 µM) strongly inhibited HCoV-229E extracellular RNA accumulation (a measure of virion release in media) at 24 hpi (Fig. 5c). Consistent with the loss of viral RNA release, harmine addition reduced intracellular viral genomic and total RNA (comprising viral genomic and sub-genomic RNAs) abundance by >90% as measured by RT-qPCR assay (Fig. S3a and S5c) and intracellular viral nucleocapsid protein by almost 80% compared to the DMSO control (Fig. 5d). To test if the compound has a prolonged inhibitory effect on HCoV-229E replication, viral outgrowth/time of incubation assays were performed. Cells were infected with virus (input MOI of 2) for an hour, after which the virus was removed, compound added at 1 hpi, and media harvested at 24, 48, and 72 hpi to quantify viral RNA release by RT-qPCR assay. As shown in Fig. 5e, harmine treatment reduced viral RNA release in media even up to 72 hpi relative to the DMSO control. Although some viral RNA release was observed at 72 hpi, subsequent TCID_50_ assays to quantify infectious particles (24) revealed a dramatic reduction in infectious particle levels (Fig. 5f), indicating that most viral particles produced in the presence of harmine are defective.

**Fig 6:**
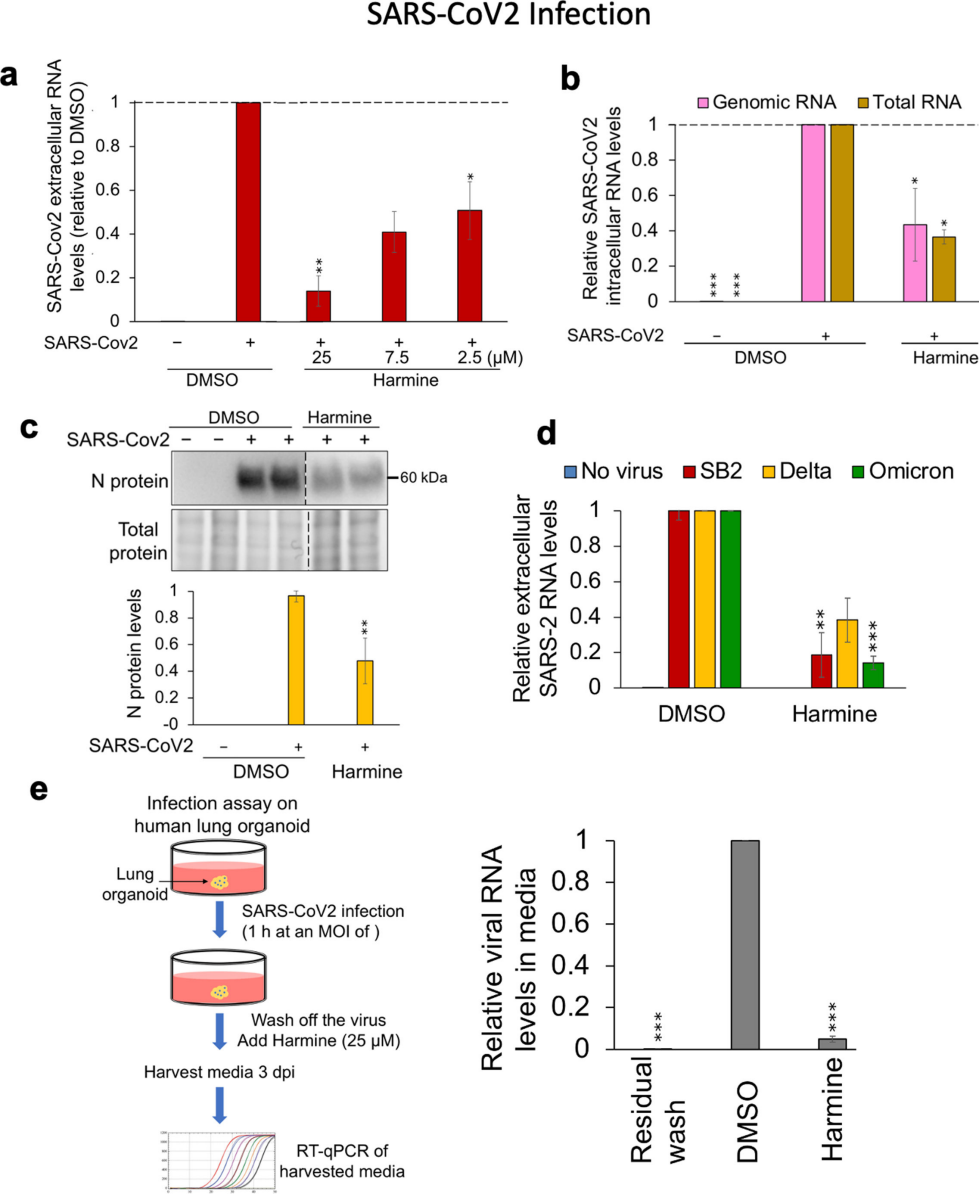
Harmine inhibits SARS-CoV-2 replication. (**a**) Huh7 cells were infected with SARS-CoV-2 at an MOI of 2 and were treated with DMSO or harmine at different concentrations (25, 7.5, and 2.5 µM). Media was harvested 24 hpi for extracellular viral RNA analysis by RT-qPCR. (**b**) Effect of 25-µM harmine on intracellular SARS-CoV-2 genomic (measured by ORF1ab primer set) and total RNA (measured by N primer set) accumulation as determined by RT-qPCR. (**c**) Representative Western blots showing SARS-CoV-2 intracellular N protein levels on the left and quantitation of the blots across three independent assays performed in duplicates on the right. Band intensities were quantified relative to DMSO control and were normalized to total protein using Bio-Rad ImageLab software. Dotted vertical lines on the blots represent cropping of lanes on the same representative blots to show harmine-treated lanes adjacent to DMSO-treated lanes. (**d**) Huh7 cells were infected with different SARS-CoV-2 variants, SB2, Delta, and Omicron BA.1 (MOI of 2), and were treated with DMSO or harmine (25 µM) 1 hpi. Media were harvested 24 hpi to measure extracellular viral RNA levels by RT-qPCR. (**e**) Effect of harmine on SARS-CoV-2 replication in human lung organoids. Lung organoids were infected with SARS-CoV-2 (SB2) for 1 h, after which organoids were washed and fresh media were added. Virus replication was monitored at day 3 post-infection by assaying the level of viral RNA in the media. Data are indicated as mean ± SD, *N* = 3 independent replicates in duplicates. *P ≤ 0.05, **P ≤ 0.01, ***P ≤ 0.001.

Success against the seasonal coronavirus 229E led us to test whether the compound has a similar ability to inhibit pathogenic SARS-CoV-2 replication. As shown in Fig. 6a, harmine treatment of SARS-CoV-2 (SB2 strain) infected Huh7 cells reduced viral RNA release in media in a dose-dependent manner (IC_50_ ~7.5 µM). Consistent with the reduction in SARS-CoV-2 RNA release, the compound also decreased intracellular SARS-CoV-2 genomic RNA accumulation (measured by amplification of ORF1ab region) by 60%, total viral RNA accumulation by ~65% (Fig. 6b), and N protein levels by 60% (Fig. 6c). Subsequent tests confirmed that harmine suppressed replication of multiple SARS-CoV-2 strains (SB2, Delta, and Omicron BA.1) (Fig. 6d), confirming its efficacy against a broad spectrum of coronaviruses. To validate harmine’s anti-viral potency in the context of a system more representative of the normal target tissue, we measured its effect on SARS-CoV-2 replication in human lung organoids (21, 48). Derived from a human embryonic stem cell line (H9), the organoids have characteristic organization of cells seen in primary tissue (49). To evaluate harmine’s effect, human lung organoids were infected with SARS-CoV-2 (SB2) at an MOI of 1 for an hour, after which the virus was removed and viral RNA accumulation in media was monitored at 3 dpi (Fig. 6e). Treatment of organoids with 25-µM harmine resulted in a dramatic reduction (~95% reduction) in virus accumulation in the media, indicating inhibition of virus replication in this tissue.

### Harmine can act late in HCoV-229E replication to inhibit viral RNA and protein production

**Fig 7:**
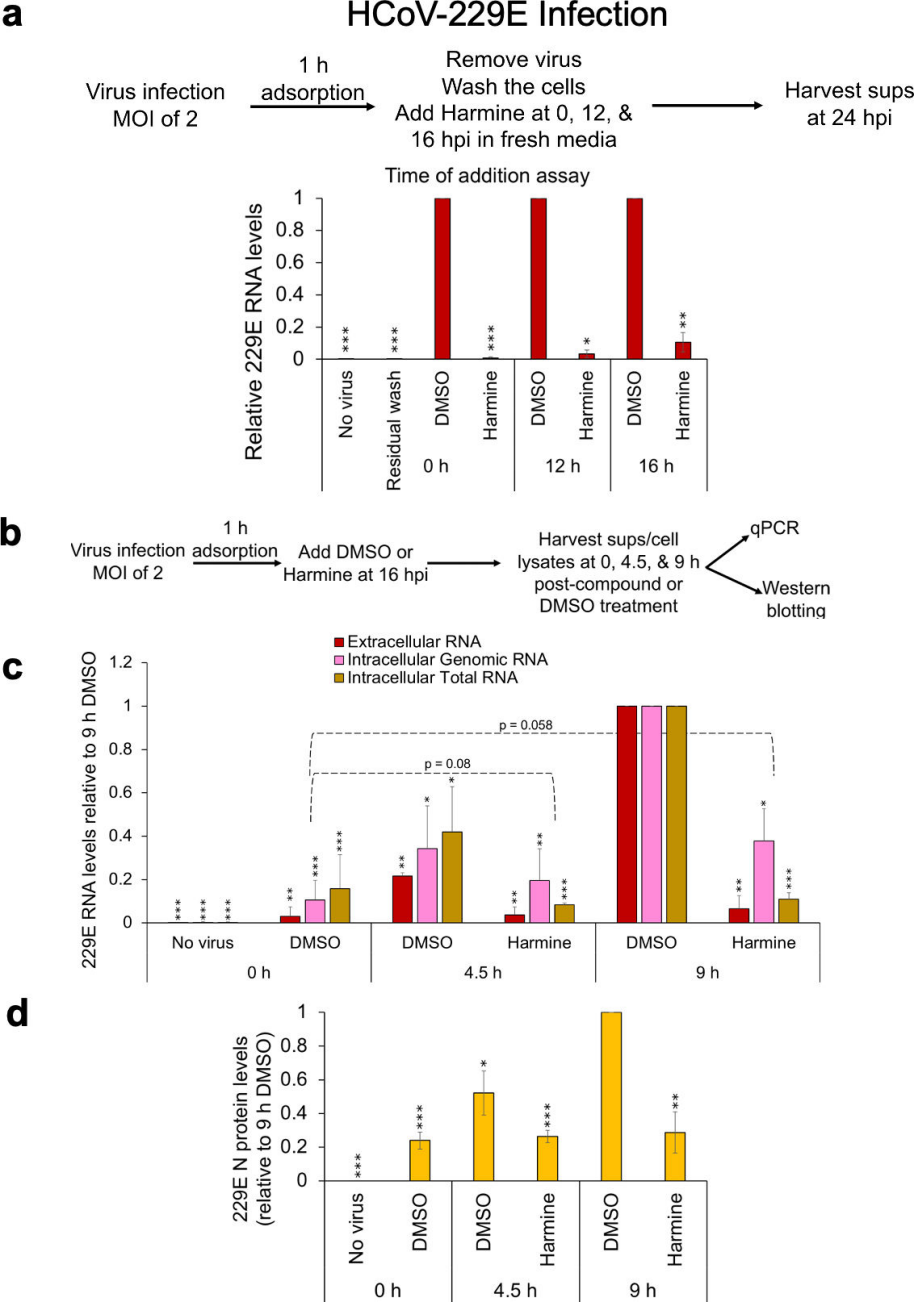
Delayed harmine addition inhibits HCoV-229E replication. (**a**) Huh7 cells were infected with HCoV-229E (MOI of 2) and treated with DMSO or harmine (25 µM) at different time points (0, 12, and 16 hpi). Media was harvested at 24 hpi for analysis of extracellular viral RNAs by RT-qPCR. (**b**) Effect of delayed harmine addition on HCoV-229E extracellular RNA release and intracellular genomic and total RNA accumulation as well as N protein accumulation 4.5 and 9 h post-DMSO or compound addition as measured by (**c**) RT-qPCR and (**d**) Western blotting, respectively. (**c**) Quantitation of viral extracellular and intracellular genomic and total RNA levels. (**d**) Quantitation of N protein by Western blotting across three independent assays performed in duplicates. Data are indicated as mean ± SD. *P ≤ 0.05, **P ≤ 0.01, ***P ≤ 0.001.

To gain insight into the stage of coronavirus replication impacted by harmine, we performed TOA assays (50). Previously, our group determined that HCoV-229E N protein expression is first detected at 12 hpi, after which N abundance dramatically increased along with appearance of viral RNA in the media (5). To determine if harmine could inhibit HCoV-229E replication after entry and initiation of viral RNA/protein production, cells were infected with virus, and DMSO or harmine was added at different times post-infection (0, 12, and 16 hpi). Media harvested at 24 hpi was used to measure effects on virus release. As shown in Fig. 7a, harmine addition at either 12 or 16 hpi is almost as effective as addition immediately post-infection in reducing HCoV-229E replication, consistent with the compound acting as a post-entry inhibitor of virus replication.

To define how virus replication was being affected, we evaluated the effect of delayed harmine addition on intracellular viral RNA and N protein accumulation. As detailed previously (5), Huh7 cells were infected with HCoV-229E (MOI 2), washed to remove residual virus, then incubated for 16 h prior to DMSO or harmine addition. As shown in Fig. 7b, delayed harmine addition only partially reduced (~2-fold) viral genomic RNA accumulation over the next 9 h. In contrast, we observed no increase in total viral RNA and N protein abundance over the same time period (Fig. 7b; Fig. S3). The primer set used for total viral RNA detection (targeting the region coding for N) detects both viral genomic RNA and all the viral subgenomic mRNAs that encode the viral structural proteins (Fig. S3a). Consequently, the differential effect of harmine on viral total mRNA vs genomic RNA accumulation suggests that the compound affects the production or stability of the viral subgenomic RNAs.

To investigate the effects of the compound on virus replication at the sub-cellular level, we also looked for changes in formation of viral replication centers as well as genomic and total viral RNA sub-cellular distribution by immunofluorescence and fluorescent *in situ* hybridization. During the process of replication, coronaviruses form replication complexes that can be detected by staining for the double-stranded RNA replication intermediates (51) within double-membrane vesicles (52) localized in perinuclear foci along with N protein (53). Using immunofluorescence staining, we tested whether delayed addition of harmine (16 hpi) altered localization of either dsRNA and/or N protein that would indicate disruption of the replicase complex. Comparison of dsRNA or N protein distribution of DMSO and harmine-treated samples fixed 4.5 and 9 h post-compound addition revealed no marked differences in the staining pattern observed (Fig. S4a). Similar examination of viral genomic and total RNA localization by FISH also did not reveal any significant changes in genomic or total RNA localization patterns in harmine vs DMSO-treated samples (Fig. S4b). Together, these data suggest that the anti-viral activity of harmine is not associated with a disruption of the viral replication complexes but rather a change in the properties of the viral RNA polymerase or differential effects on viral RNA stability.

### Harmine has limited effect on the host cell transcriptome, many of the affected events overlapping with those altered by 1H3 treatment

**Fig 8:**
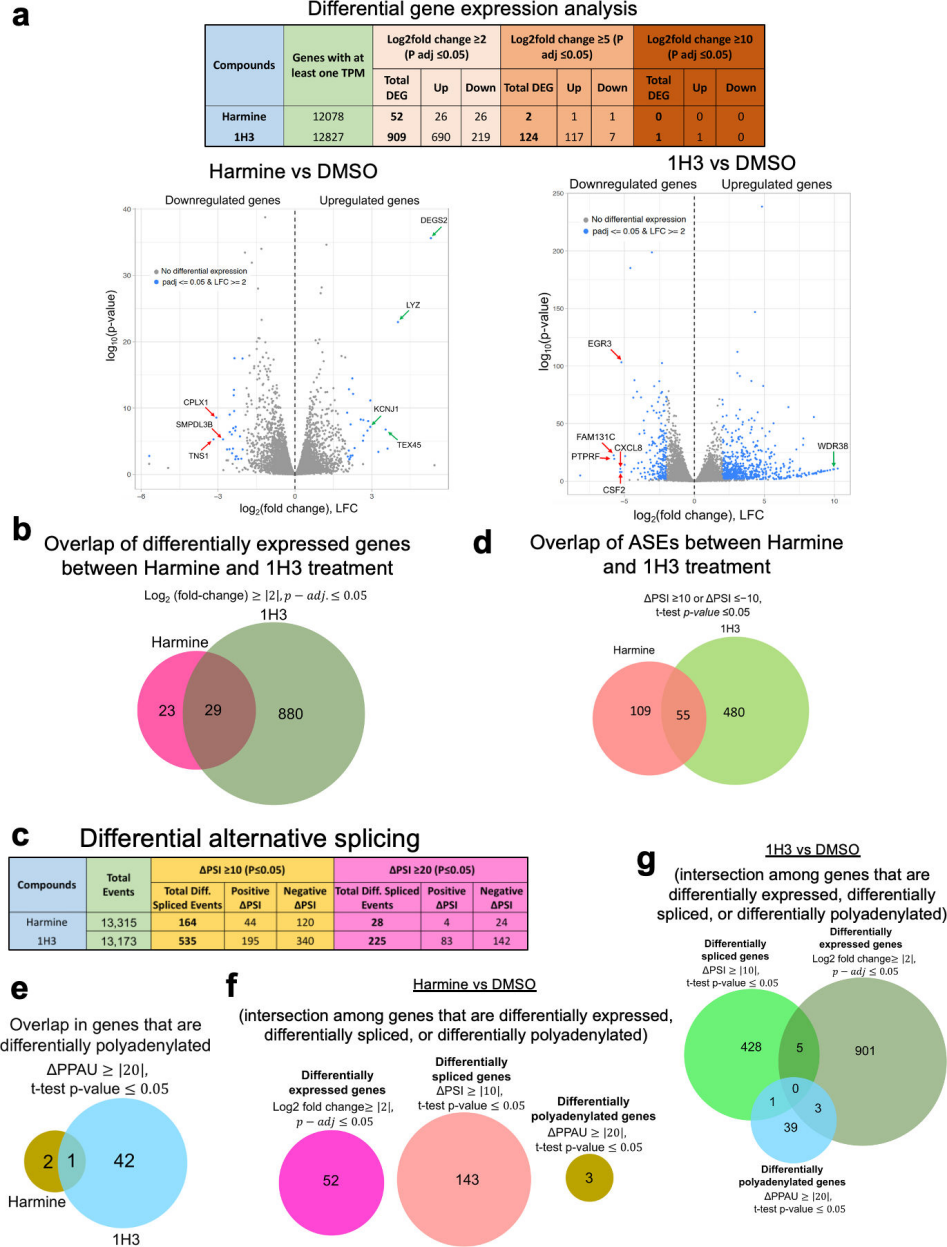
Harmine has limited effect on the host transcriptome, many of the affected events overlapping with those altered by 1H3 treatment. (a) Table showing global profiling of host RNA expression for harmine vs DMSO and 1H3 vs DMSO treatments as assessed by RNA-Seq. On the bottom to the left and the right are the volcano plots showing differential gene expression analyses. Blue dots represent the genes that are differentially expressed, and gray dots represent the genes with no differential expression at |LFC| adjusted *P* value of ≤ 0.05. The genes whose differential expression have been validated by RT-qPCR are shown on the volcano plots by arrows. (b) Venn-Diagram showing overlap of genes whose expression at the level of RNA abundance is impacted by harmine and/or 1H3. (c) Table showing global profiling of host ASEs, represented by changes in percent spliced in values (ΔPSI) out of total events detected by RNA-Seq. (d) Venn diagram showing ASEs affected by harmine and/or 1H3. (e) RNA-Seq was used to examine for changes in alternative polyadenylation (APA) site usage upon treatment with either harmine or 1H3. Shown in the Venn diagram are the number of APA events altered [at ∆PPAU> (20)] and the overlap of events affected by either harmine or 1H3 addition. (f, g) Extent of overlap of genes that are differentially expressed, differentially spliced, or differentially polyadenylated upon treatment with (f) harmine or (g) 1H3.

Given harmine’s ability to inhibit replication of two unrelated viruses (HIV-1 and coronaviruses) without significant cell toxicity, we explored how the compound impacts the host cell. In addition, given the ability of both harmine and 1H3 to inhibit HIV-1 replication, but that only the former inhibited coronaviruses, we looked for similarities and differences between these two compounds. To probe these questions, we examined changes in gene expression, alternative splicing, and alternative polyadenylation in primary CD4^+^ T cells treated with DMSO, harmine, or 1H3 over a period of 3 days using RNA-Seq. Gene quantification analysis detected 20,008 protein-coding genes. However, only genes with expression levels higher than one transcript-per-million reads in any one treatment group were evaluated to ensure greater reproducibility between samples. Differential gene expression analysis of compound-treated and DMSO-treated donor samples (average read counts from two donors) using the DSeq2 package identified multiple genes positively or negatively modulated using the criteria of at least log_2_ fold change of ≥2 (|LFC| ≥2), and with a maximum corrected *P* value of 0.05. Analysis (shown as a volcano plot in Fig. 8a and listed in Table S4) revealed that, of the 12,078 genes meeting the cutoff criteria (greater than one TPM), harmine treatment altered expression of 52 genes (26 upregulated and 26 downregulated) at |LFC| of ≥2 and only two genes at |LFC| of ≥5, with a *P* value of ≤0.05, indicating limited alterations in host RNA abundance. For 1H3-treated samples, of 12,827 genes that met the cutoff criteria, expression of 909 genes (690 upregulated and 219 downregulated) were differentially altered at |LFC| of ≥2, 124 at |LFC| of ≥5, with a *P* value of ≤ 0.05, and one at |LFC| of ≥10, with a *P* value of ≤ 0.05 (shown as a volcano plot in Fig. 8a and listed in Table S4). Genes whose expression were most significantly altered (LYZ, KCNJ1, TEX45, DEGS2, CPLX1, SMPDL3B, and TNS1 in harmine-treated samples and WDR38, EGR3, FAM131C, CXCL8, PTPRF, and CSF2 in 1H3-treated samples) were validated by RT-qPCR across two to four donor samples (Fig. S5a and b). Using a cutoff of |LFC| of ≥2, 56% of the genes (29 genes) altered upon harmine addition were also affected by 1H3, all in the same direction (upregulated or downregulated) (Fig. 8b; Table S4).

Given the effects of both harmine and 1H3 on SR kinase expression and the role these kinases play in regulating alternative splicing, we also explored how AS patterns were altered by either treatment. Changes in AS were evaluated by quantifying all the alternative splicing events using the percent spliced-in (PSI) metric (54), the percentage of long isoform over total isoforms present (54), excluding events whose average short plus long isoform expression was below 2 TPM. Of 13,315 events meeting the criteria for DMSO vs harmine, 164 events from 143 genes displayed an absolute difference in PSI value (|ΔPSI|) of ≥10% and *t*-test adjusted *P* value of ≤0.05, 1.2% of the total events detected. Only 28 events showed altered splicing patterns at |ΔPSI| of ≥20% and *t*-test adjusted *P* value of ≤0.05, indicating limited alterations in the splicing pattern upon harmine addition (Fig. 8c). Similarly, for DMSO vs 1H3, 535 events (434 genes) were differentially spliced at |ΔPSI| of ≥10% and 225 events at |ΔPSI| of ≥20% and *t*-test adjusted *P* value of ≤0.05, of 13,173 events analyzed (Fig. 8c). SESs were the most common for both harmine and 1H3 treatments, representing 49% and 44%, respectively, of the total changes in ASEs identified by RNA-Seq (Fig. S6a). Shown in Fig. S6b are the scatter plots indicating changes in the splicing patterns of the genes/events for harmine vs DMSO and 1H3 vs DMSO treatments, respectively. Of the 164 events altered by harmine, 55 were common with 1H3, 54 of which were modulated in the same direction, indicating a high degree of similarity in response to these treatments (Fig. 8d). A complete list of the differential ASEs observed upon harmine or 1H3 treatment and the list of events common between the two treatment conditions are shown in Table S5. ASEs for some of the most perturbed genes affected by harmine or 1H3 treatment were validated by RT-PCR (Fig. S6c). GO enrichment analysis (using gProfiler; https://biit.cs.ut.ee/gprofiler/gost) (55) of the differentially spliced genes for harmine-treated samples showed enrichment for factors involved in regulation of NF-kappaB (NF-kB) signaling, RNA stability, and factors involved in the cellular stress response (Fig. S7a). Interestingly, previous studies have reported harmine’s ability to inhibit replication of viruses such as enterovirus 71 and HSV-1 and HSV-2 through suppression of the NF-kB pathway (56 – 58). During manual curation of the splicing events affected by harmine, we noted changes in splicing patterns of several host RNA processing factors, including the SR kinase CLK1 as well as the SR proteins SRSF2 and SRSF3 (Fig. S7b). Similar manual curation of the splicing events affected by 1H3 showed changes in splicing patterns of RNA processing factors including CLK1, DYRK1A, SRSF2, SRSF3, SRSF6, and Tra2ß (Fig. S7b).

Like AS, APA can also significantly impact the fate of mRNA through loss or gain of 3′ UTR sequences that contain cis-elements recognized by microRNAs (miRNAs) and/or RNA-binding proteins (RBPs) (59). Through targeting of miRNAs or binding to RBPs, the 3′ UTRs regulate mRNA stability, translation, export, and sub-cellular localization (33). Given the role of several SR proteins in regulating poly(A) site selection (60 – 62) and alteration of select SR protein abundance with harmine or 1H3 (16) treatment, we examined for changes in alternative poly(A) site usage in CD4^+^ T cells treated with DMSO, harmine, or 1H3 from the RNA-Seq data using QAPA software (30). Of 14,342 protein-coding genes detected using the QAPA pipeline, 6,935 genes were identified to have only one poly(A) site and were filtered out from the analysis. From the remaining 7,407 genes containing at least two poly(A) sites, we focused on the gene set containing only two known poly(A) sites. Of those 2,715 genes with two poly(A) sites, we focused on a set of 1,466 proximal 3′ UTR events with at least 2 TPM of any isoform in either data set. Of 1,466 genes, harmine treatment altered PAU of only three genes, SUCLA2, RTL8A, and CD68, at |∆PPAU| of ≥∆20% and *t*-test *P* value of ≤0.05. Two of the three genes, SUCLA2 and RTL8A, favored usage of proximal poly(A) site upon treatment with harmine as demonstrated by highly positive ∆PPAU values of 35.8 and 34.3%, respectively, shortening the 3′ UTR, while harmine favored distal PAU for CD68 as observed by a highly negative ∆PPAU value of −33.7%, compared to the control, lengthening the 3′ UTR. Similarly, 1H3 treatment altered PAU for 43 of the 1466 genes examined, at |∆PPAU| of ≥20% and *t*-test *P* value of ≤0.05, 4 of those favoring proximal PAU and the remaining 39 favoring distal PAU. As indicated in the Venn-Diagram (Fig. 8e), comparison of genes with altered PAU upon harmine or 1H3 treatment revealed only one gene common at the level of APA regulation, i.e., CD68, and both conditions favored 3′ UTR lengthening. Table S6a and b summarize changes in proximal APA usage in harmine vs DMSO and 1H3 vs DMSO treatments, respectively. With 0.2% of changes in alternative poly(A) usage upon harmine treatment at the dose used to inhibit the virus, harmine had a very limited impact on host APA.

We then compared the differentially altered gene sets obtained from APA analysis with differentially expressed genes and differentially spliced genes upon harmine or 1H3 treatment. No overlap was seen for genes regulated at the level of RNA abundance, AS, or APA upon harmine treatment (Fig. 8f). Comparison of genes altered, upon 1H3 treatment, at the level of DGE, AS, and APA revealed only a small number of genes regulated at two different levels (DGE and AS, DGE and APA, or AS and APA) (Fig. 8g). Five genes (CREM, DDIT3, IRF1, P4HA1, and PHF1) were regulated both at the level of RNA expression and AS (Table S5). Likewise, three other genes (CAVIN3, N4BP3, and CSKMT) were regulated at the level of differential gene expression as well as APA. Of those, CAVIN3 and N4BP3 favored 3′ UTR lengthening and showed downregulation of gene expression. CSKMT showed upregulation of gene expression, though it favored 3′ UTR lengthening. Following 1H3 treatment, only one gene with altered polyadenylation, TBC1D25 (favoring distal PAU), also resulted in differential splicing (promoting exon exclusion). The minimal impact of harmine on the host transcriptome at doses sufficient to suppress HIV-1 replication highlights the greater sensitivity of this virus to changes in activity of cellular RNA processing factors. The high degree of overlap in the effects seen upon harmine or 1H3 addition suggests shared mechanisms or pathways affected by both compounds, indicating a certain degree of similarity in their biological effects on the host cell transcriptome.

## DISCUSSION

The ability of viruses to develop resistance to current therapeutics, coupled with the emergence of diverse viral pathogens with pandemic potential, calls for the identification of compounds able to inhibit a broad array of viruses (1). Unlike bacteria, fungi, and other parasites which offer a diverse number of targets for drug development, viruses, due to their compact genome and relatively simple anatomy, offer fewer druggable targets (generally the virus-encoded polymerase or protease) (1). Given the requirement of viruses to encode multiple proteins from their limited genome size, many viruses rely on alternative splicing for their replication (2, 63). Interestingly, many RNA viruses that replicate within cytoplasmic factories, independent of splicing, also have a reliance on host splicing factors (14, 15). Examples include the T1L protein of reovirus, which alters SRSF2 sub-cellular localization, impacting host pre-mRNA splicing to enhance virus replication (64). Alternatively, host splicing factors often have functions in the cytoplasm which may contribute to viral RNA or protein expression (6).

In the present study, we assessed several host SR kinase inhibitors for anti-viral activity, but only harmine inhibited two unrelated viruses: HIV-1, which critically relies on the host alternative splicing for its replication (2), and coronaviruses (HCoV-229E and SARS-CoV-2), whose life cycle takes place in the cytoplasm, and genome replication/expression does not directly require RNA splicing (65). Harmine also inhibits replication of several other viruses, including enterovirus 71 (56), herpes simplex virus (57), influenza A virus (IAV) (66), and dengue virus (67), supporting the potential of harmine, or related compounds, as possible host-directed broad-spectrum anti-virals. The difference in the ability of the SR kinase inhibitors tested to inhibit HIV-1 and coronavirus replication indicates the observed response is not due to a general reduction in SR protein phosphorylation but rather to changes uniquely induced by harmine.

Harmine inhibited HIV-1 replication/gene expression in multiple different cell systems including HeLa B2, CEM-HIV, and primary CD4^+^ T cells infected with replication competent HIV-1 without any cytotoxic effects. The compound’s reduction of HIV-1 Env gp160, Gag, and Tat protein levels was correlated with loss of viral US and SS RNA accumulation but a more limited impact on MS RNA abundance. Similar to another SR kinase inhibitor, 1H3 (16), harmine induced limited changes in HIV-1 splice-site usage within the MS RNAs, suggesting that it affects the extent of primary HIV-1 transcript splicing rather than selection of individual splice sites. Although the compound yielded only a limited reduction in the spread of infection among primary macrophages, unlike Epivir/Kaletra, it reduced intracellular Gag levels in already infected cells (as measured by Gag MFI), consistent with a decrease in HIV-1 US RNA accumulation. The reduced viral antigen load in infected cells could be beneficial as the presence of such viral antigens can contribute to the chronic immune activation present during infection (68).

To shed light into the mechanism underlying harmine’s inhibition of HIV-1, we investigated the roles of its known targets, DYRK1A and MAO A. We initially hypothesized that loss of DYRK1A function mediated the anti-viral effect of harmine. However, DYRK1A depletion by 80%–90% resulted in no change in HIV-1 expression and did not affect harmine’s capacity to inhibit HIV-1. Studies have shown an even greater potency of harmine against MAO A (IC_50_ = 5 nM) (69). However, other MAO A inhibitors, harmane (IC_50_ = 500 nM [70]) and moclobemide (IC_50_ = 6.1 µM [71]), did not suppress HIV-1 expression, indicating that harmine’s anti-HIV-1 activity is mediated through an effect on other host factors. Although harmine is described as an inhibitor of DYRK1A, a kinome scan indicated that it can inhibit multiple SR kinases including CLK1, 2, and 4 (46). In the case of CLK1, inhibition of the enzyme’s activity increases its expression due a negative feedback loop in which CLK1 auto-regulates the splicing of its mRNA (37, 72). Consistent with an inhibition of CLK1 function, harmine (or 1H3) (16) addition increased CLK1 RNA splicing by promoting exon 4 inclusion (confirmed by RT-PCR, Fig. S6b) and reducing retention of intron 4 (observed through RNA-Seq), which increases production of catalytically active CLK1 (72) protein levels, similar to that observed upon treatment with the CLK1/CLK4 inhibitor, TG003 (36, 72) (Fig. 4a). However, unlike TG003, both harmine and 1H3 inhibit HIV-1 Gag expression and reduce CLK2 levels. Alteration of CLK1 and CLK2 expression levels by harmine and 1H3 is also comparable with another anti-HIV-1 compound, 1C8 (73), known to affect CLK activity (16). Our previous demonstration that CLK1 or CLK2 depletion have opposing effects on HIV-1 gene expression and that a 50% decrease in CLK2 levels reduces HIV-1 gene expression (16) supports the hypothesis that altering the relative abundance of individual CLK kinases might be responsible for the underlying response to harmine addition. Consistent with the changes in SR kinase expression, harmine altered abundance of select SR proteins, whose activity and localization are dependent on their state of phosphorylation by the SR kinases (74). Harmine treatment of primary CD4^+^ T cells reduced levels of SRSF1, 3, 4, and 7 and Tra2β while inducing a slight increase in SRSF9 levels. A similar reduction of SRSF3 and 4, and Tra2β, and an increase in SRSF9 levels was also observed upon 1H3 addition (16). Consistent with the changes in SRSF3 and Tra2β levels affecting HIV-1 expression, we note that activation of primary CD4^+^ T cells, generating a cell state permissive to HIV-1 replication (75), increased levels of SRSF3 and Tra2β (16). Furthermore, changes in SRSF3 or Tra2β expression/activity do affect HIV-1 RNA processing and expression (4, 76). Review of the RNA-Seq data determined that changes in SR protein expression were not due to alterations in the abundance of their corresponding mRNAs or APA usage. However, harmine or 1H3 treatment did change the splicing patterns of some SR protein mRNAs as demonstrated by RNA-Seq. Harmine treatment altered splicing of SRSF2 and 3, while 1H3 altered splicing of SRSF2, 3, 5, and 6 and Tra2ß. Together, these data indicate that the selective changes in SR protein levels induced by these compounds could be due to alterations at the level of RNA splicing and/or protein synthesis/stability. A recent study has demonstrated that SR protein expression levels are controlled by alternative splicing coupled with non-sense-mediated decay of their poison exons (77). Poison exons are ultra-conserved regions in SR protein genes that contain non-coding exonic sequences, which, when included, introduce a premature termination codon and target the mRNA for degradation (78).

In addition to the similar changes in SR kinase and SR protein expression with either harmine or 1H3 addition, we also observed a high degree of overlap in host genes affected by either compound, both at the levels of RNA abundance and differential splicing; 56% of the genes affected by harmine at the level of RNA abundance were common to 1H3, all the changes (upregulation or downregulation) being in the same direction. Similarly, 34% of the alternative splicing events affected by harmine were common to 1H3, and 98% of those were altered in the same direction. Together, the strong similarity between the two compounds in how they affect gene expression and splicing suggests a common mechanism for their suppression of HIV-1 gene expression. In contrast, harmine had a very limited impact on APA (only three of 1,466 genes with altered PAU), and only one of those three were common with 1H3’s APA regulation. Together, these findings suggest that harmine’s unique ability to inhibit HIV-1 and coronavirus replication, among the SR kinase inhibitors tested (including 1H3), is due to its differential modification of factors/processes critical to the replication of this group of viruses.

Harmine exhibited pan anti-coronavirus activity, inhibiting replication of a seasonal coronavirus (HCoV-229E) and three different pathogenic variants of SARS-CoV-2. The compound was active against coronavirus not only in a cell line but also in a human lung organoid model that captures the physiologically relevant context of human SARS-CoV-2 infection (21). Consistent with the reduced coronavirus particle release in media, harmine suppressed viral intracellular genomic and total RNA accumulation in agreement with the loss of viral N protein synthesis. The ability of harmine to inhibit coronavirus replication upon its addition at 16 hpi indicates action at a post-entry stage of virus replication. Similar to its differential effect on the abundance of HIV-1 RNAs (US, SS, and MS), harmine selectively altered the accumulation of HCoV-229E viral total vs genomic RNAs when added at 16 hpi. While some reduction (~2 fold) in viral genomic RNA accumulation was observed 9 h post-harmine addition compared to DMSO, total viral RNA abundance (comprising genomic and sub-genomic RNAs) did not increase across different time points post-compound addition. Harmine addition had limited effect on dsRNA staining patterns or viral genomic RNA accumulation in perinuclear foci, indicating no change in the formation/stability of viral replication complexes. If viral replicase formation is unaffected, why do we see the selective loss of total viral RNA accumulation upon harmine addition? To express their full coding capacity, coronaviruses generate multiple sub-genomic RNAs encoding the viral structural proteins (79). In contrast to synthesis of the viral genomic RNA (involving a direct copying of the viral template), these sub-genomic mRNAs are a nested set generated by discontinuous movement (jumping) of the viral polymerase on the template (80). Harmine’s inhibition of HCoV-229E viral total RNA accumulation suggests a preferential inhibition of sub-genomic mRNA synthesis or reduction in their stability.

Although a direct requirement of splicing in coronaviruses has not been shown, several studies have demonstrated interaction of coronavirus RNA, including SARS-CoV2 RNA, with many host splicing factors including hnRNPs and SR proteins, suggesting a role in controlling coronavirus replication (81, 82). In addition, the N protein of all coronaviruses contains a highly conserved arginine/serine-rich motif (14) that has been shown to be phosphorylated by host SR kinases, either GSK3α/β or SRPK1 (15, 83, 84). The phosphorylation of viral N protein modulates its interaction with the viral RNA genome and recruitment of host proteins required for viral replication (14, 15). In the context of the mouse hepatitis virus (MHV) JHM strain (JHMV), site-specific phosphorylation of N by GSK3 is critical for the recruitment of the host helicase DDX1 to the viral replicase complex to enable the transition from discontinuous to continuous transcription of the viral genome (85, 86). In our studies, Western blots of coronavirus-infected cells treated with harmine did not reveal any shift in N protein migration (Fig. 6 and 7), indicative of changes in phosphorylation. However, harmine could have induced site-specific changes in N phosphorylation (not easily detected by Western blots) that could impact the function of N protein and its interaction with the viral RNA.

Dependence of viruses on the host splicing machinery is not unique to the viruses examined in this study. Hepatitis B virus does not require splicing of its RNA but has a viral core protein rich in SR/RS-dipeptides that is a substrate for SRPK1, modification of which impacts its binding to RNA and capsid assembly (87). IAV, another RNA virus, replicates in the nucleus and requires splicing of a subset of its transcripts (88). Given this dependence on splicing and host SR proteins (mainly SRSF2, 3, and 5), IAV replication can be blocked by addition of the CLK inhibitor, TG003 (6, 89). SR kinases are also important for replication of several other viruses, including adenovirus, human cytomegalovirus, HSV, human papilloma virus, hepatitis C virus, Ebola, and Sindbis virus (6). Despite differences in the underlying mechanisms by which these viruses replicate, it is clear that many viruses have evolved to depend on SR kinase activity and/or the host factors they modify (6). Consequently, appropriate alteration of SR kinase activity could serve as a basis for the development of broad-spectrum anti-virals against which viruses may not easily mount a resistance. Our RNA-Seq data demonstrate that, at a dose (10 µM) required to significantly reduce HIV-1 RNA accumulation, harmine had limited impact on the host cell transcriptome, as measured at the level of DGE (0.43% of genes detected), alternative splicing (1.2% of the events detected), and APA (0.2% of the genes/events measured). These observations further highlight the sensitivity of viruses to small changes or perturbations in host factor activity, opening up a new avenue to manipulate virus growth with minimal impact on the host.

## SUPPLEMENTAL MATERIAL

Supplemental tables and figures jvi.00396-23-s0001.pdf Tables S1 to S3 and Fig. S1 to S7.

Table S4: DGE analysis jvi.00396-23-s0002.xlsx RNAseq differential gene expression.

Table S5: PSI analysis jvi.00396-23-s0003.xlsx RNAseq differential splicing analysis.

Table S6: PPAU analysis jvi.00396-23-s0004.xlsx RNAseq differential polyadenylation analysis.

ASM does not own the copyrights to Supplemental Material that may be linked to, or accessed through, an article. The authors have granted ASM a non-exclusive, world-wide license to publish the Supplemental Material files. Please contact the corresponding author directly for reuse.
