# Supplementary material for: On a path toward a broad-spectrum anti-viral: inhibition of HIV-1 and coronavirus replication by SR kinase inhibitor harmine
Source: J Virol. 2023 Sep 14;97(10):e00396-23. doi: 10.1128/jvi.00396-23 (PMC10617549; doi:10.1128/jvi.00396-23)
Supplement: Supplemental tables and figures — Tables S1 to S3 and Fig. S1 to S7. [file jvi.00396-23-s0001.pdf]

Table S1 Primary antibodies used in the study					
**Antibody**	**Type**	**Size (kDa)**	**Dilution**	**Company**	**Catalog No.**
Gag p24	Ms	55, 41, 24	1:500 in 1X TBST	NIH AIDS Reagent	#1513
Env gp41	Hu	160	1:1000 in 1X TBST	NIH AIDS Reagent	#531
Tat 1D9	Ms	16, 14	1:300 in 1X TBST	NIH AIDS Reagent	#7383
DYRK1A	Rb	90	1:1000 in 1 X TBST	Cell Signaling Technologies	#8765S
GAPDH	Rb	35.8	1:3000 in 1X TBST	Sigma-Aldrich	G9545
Tubulin	Rb	55	1:3000 in 1 X TBST	Cell Signaling Technologies	#2144S
CLK1	Ms	60	1:1000 in 5% BSA-TBST	Santa Cruz	#515897
CLK2	Rb	60	1:1000 in 5% BSA-TBST	Abcam	ab65082
CLK3	Rb	60	1:1000 in 5% BSA-TBST	Abnova	H00001198-M05
SRPK1	Rb	110	1:2000 in 5% BSA-TBST	Cedarlane	OAAN01583
SRSF1 (ASF/SF2)	Ms	32	1:1000 in 1X TBST	Life Technologies	32-4500
SRSF2 (SC35)	Rb	~35	1:1000 in 1X TBST	Abcam	ab11826
SRSF3 (SRp20)	Ms	20	1:1000 in 1X TBST	Life Technologies	33-4200
SRSF4 (SRp75)	Rb	75	1:2000 in 1X TBST	Novus Biologicals	NBP2-04144
SRSF5	Rb	~40	1:1000 in 1X TBST	MBL	RN082PW
SRSF6 (SRp55)	Rb	55	1:2000 in 1X TBST	Novus Biologicals	NBP2-04142
SRSF7 (9G8)	Rb	~35	1:2000 in 1X TBST	Abcam	ab137247
SRSF9 (SRp30c)	Rb	27	1:1000 in 1X TBST	MBL	RN081PW
SRSF10 (FUSIP1)	Ms	37	1:1000 in 1X TBST	Novus Biologicals	H00010772-M07
Tra2ß	Rb	~40	1:1000 in 1X TBST	Abcam	ab31353
Feline anti-coronavirus antibody (FIPV3-70 for HCoV-229E N)	Ms	~60	1:4000 in 1 X TBST	Novus Biologicals	NB10064754
SARS-CoV2 N	Rb	~60	1:4000 in 1 X TBST	Sino Biological	40143-R019

Table S2. Sequences of HCoV-229E RNA FISH probes used in the study.

229E Genomic RNA FISH p	Sequence
1	acgtcaaatgcacggacaca
2	ggacttcagattttttccga
3	tagaacgcgtcatccttatc
4	cgtggtccataactgactta
5	cagcattacagcctttaagt
6	ccctcatgccaagtaaagaa
7	ttacgcagagcgaagcacaa
8	gtgtatgtcctcattttcta
9	accttacctaaagcagcata
10	gcaccatttcgtcgcaaaaa
11	atcttggttgtctaaggtca
12	tatgacgtgcagtagggtat
13	tacccataacaggcatcata
14	aaacctccttatgttctgtg
15	ctgtccccaatacttaaagt
16	tctcgtcatggcaatcaaca
17	tggttgcgaagagtgtgtta
18	cacttttctgcatagaggtc
19	gtaaccagcagtagcaacta
20	gcgttggatctgtcacaaac
21	gggatgttaatcctgtactc
22	aaatggccaggttttactgt
23	ctcatcgaaaaacccctgag
24	ccaatatccagcatagtagg
25	aaaatagcgagctgccactt
26	ctagatgtaatacagccacc
27	ccaaccagcgcttttattaa
28	atagcatcttgttcctcata
29	cttaccagatatggcgtatt
30	gtcttgtagtcatagtagct
31	gcatttctggtagctactat
32	cttggtagtgccgataacaa
33	tttaacatattatcccaccc
34	gaatcattgagggcatagct
35	cctaagatcatagccgacaa
36	tagccgtacaacatgtgaca
37	agttgcatcaccagaagttg
38	tcagcaatgtaaccaagtcc
39	agtccaacacttagctgtac
40	aatctgcatagtgtgctgtg
41	gctaggatctggatatggta
42	gacagcatcagtcttagtga
43	gccagagaaacatagcgttc
44	agacaatgggtaggcatcta
45	tgtactcaggtttaggatgc
46	ttgacccagtctaacaatgc
47	ccttcgttaagagtcttgtt
48	gcataaaagctttcatccca

HCoV 229E Total RNA FISH pr	Sequence
1	atcagcccatttgactgtag
2	gaccacgttgtggttcagat
3	cttccaaggttgttcactat
4	tgggtaccaaattacgaggt
5	ccctttctagttctgaaacg
6	gtcctgtgccaagataataa
7	gctctctaaattttgcatct
8	aacccagacgacaccttcaa
9	cagttttagcaccatcaaca
10	ctaacaccgtaacctgtagg
11	ctctggttctgaattcttgc
12	gcttttgattgaagtgtggt
13	cttcaacaacagtaacacca
14	gaaggagcacgggagtcagg
15	tctgcgacctcgactgagac
16	gattgaggtttggattcacc
17	tctgtcacttgaaggattcc
18	tttaagagccgcagcaactg
19	gaggcttgtcaaaacctaaa
20	cgagaaggcttaggagtacc
21	cacatcatcattaggctgtc
22	gggccaaaacattgtgtgac
23	tttaacaccattggccacaa
24	ggcacaagctcagcaaattg
25	tcaaacagcatagcagctgt
26	tgcctgactctttggaaaca
27	gtgaaagtcaagaccacagt
28	tttgggcacagtcactctag
29	attctagtgcactagggtta
30	cagttgcaggtgaagtttga
31	aaacttcatcacgcactggt
32	gcaatgtattcatctacggc
33	ccatcatacatagatcagag
34	attaaaagcagctctgatcg
35	agccaaagcaagggatcatg
36	gtataagactagatccttgt
37	actaccactggcttaccatt
38	gtagcaaatttcttatacct
39	gttcacctaggttcagtaac
40	ctccagcacatttgtaatga
41	ctcgtcaatgcgatctttga
42	tgactggctcttccattgtt
43	actagataggtctcaagaca
44	atcgaaaccgttccattagc

Table S3. Primers for validation of RNA-Seq Data

Gene	F primer	R primer	
DEGS2	CGGCGCAAGGAGATACTGG	GTTGTGCGAGATGTCGTGGA	Primers for DGE Validation
LYZ	GGCCAAATGGGAGAGTGGTTA	CCAGTAGCGGCTATTGATCTGAA	
TEX45	GGCAGTCTGGACACCTTCAT	CATCTCTTCAGTCACCGGGG	
KCNJ1	GTGGAGGCACAGTCAAGGTTT	GCTACTGCATACCACAGGAGAC	
SMPDL3B	GGTATCCAAAGACCCCTTCCA	CACCAGTCCAGAGAATGAAGTC	
CPLX1	CAACTCCGAGGGGAGCTTG	GGCAGGTACTTGATGACGGTG	
TNS1	GTACGTCACAGAGAGGATCATCG	GCAGGTAGTTGCCTCCATGTT	
WDR38	GGGATGTGGCAAGAGCGAA	GGTGCCCAACTAAGAGGCG	
FAM131C	GCTACCTGTCCGACTCCAGAT	GCAGCAGTAATGCTCGTCCT	
EGR3	GACATCGGTCTGACCAACGAG	GGCGAACTTTCCCAAGTAGGT	
CSF2	TCCTGAACCTGAGTAGAGACAC	TGCTGCTTGTAGTGGCTGG	
PTPRF	GCTTCGAGGTCATTGAGTTTGA	CCCATGTCGATGGAAGGGAA	
CXCL8	GTGCAGTTTTGCCAAGGAGT	CTCTGCACCCAGTTTTCCTT	

Gene	F primer	R primer	
ABHD5	TGCAGCGTTTAAGGCCTGAT	CCCATCAGAGCTTCAGTGTGT	Primers for RT-PCR V
RPS24	GGAACGCAAGAACAGAATGAAGAA	CGAAAAATCCACAGTGGCCAC	
DOK2	GAGAAGGGAAACACACGGCT	AACCTGTAGGGCCAGTCGTA	
HNRNPH3	ACGGTATTGAGATGGATTGGGT	TCTCTCATTCTGTCATCAAAGCCA	
NAP1L14	AAGGTGACGAGGAGGGAGAA	AGATTCAGCGGCTGGAAACA	
CLK1	AGTCGACGCTACATTGATGAGT	TGCACTCCACAACTTTTCCAAAA	

Supplementary Figures

FIGURE S1. Effect of harmine on HIV-1 expression in HeLa B2 and CEM-HIV cell lines

(a) and **(b)** are the dose response curves in HeLa B2 and CEM-HIV cells, respectively. HeLa B2 or CEM-HIV cells were incubated with Harmine at increasing concentration and HIV-1 gene expression induced with Dox (4.5 µM) (for HeLa B2) or Dox (4.5 µM) +prostratin (2.56 µM) (in CEM-HIV cells) for 24 h. Cells treated with 1% DMSO with or without the inducer served as positive and negative controls, respectively. Dose response effect on HIV-1 gene expression is expressed relative to intracellular Gagp55 levels (HeLa B2) or GagGFP levels (CEM-HIV) of DMSO treated samples. Effects of Harmine on cell viability was assessed using alamarBlue assay across n=3 independent assays. **c**) RT-PCR performed on DMSO or Harmine treated cDNA samples to detect HIV-1 MS RNA species. Shown on the left is the representative polyacrylamide gel and on the right is the quantitation of n=3 independent assays performed in duplicates. Band intensities were quantified relative to DMSO control and normalized to total protein using Bio-Rad ImageLab software.

FIGURE S2. Effect of MAO A inhibitors on HIV-1 protein levels. (a) Hela B2 or **(b)** CEM-HIV cells were induced for HIV-1 gene expression and treated with DMSO, Harmine (10 µM), Harmane (10 µM), or Moclobemide (30 µM). Cells were harvested 24 h after treatment to examine HIV-1 protein levels by western blotting. Shown on the right are the quantitations of the western blots across three independent assays performed in duplicates. Band intensities were quantified relative to DMSO control and normalized to total protein using Bio-Rad ImageLab software. Data are indicated as mean ± SD, $^{**}p \leq 0.01$ and $^{***}p \leq 0.001$.

FIGURE S3. Effect of delayed Harmine addition on HCoV-229E intracellular N protein accumulation. **(a)** Schematic of coronavirus genomic RNA and sub-genomic RNAs. Arrowheads represent forward and reverse primer positions. ORF1ab primer set measures viral genomic RNA while N primer set measures total viral RNA. **(b)** Huh7 cells were infected with 229E (MOI 2) for 1 h, inoculum removed, and fresh media added. 16 hpi, media was changed to one containing DMSO or 25 μM Harmine and samples harvested immediately (t=0), 4.5, or 9 h post-DMSO or compound addition. **(c)** Representative western blots for 4.5 h (top) and 9 h (bottom) harvest post-compound treatment. **(d)** Quantitation of the western blots across three independent assays performed in duplicates. Data are indicated as mean ± SD, $*p \leq 0.05$, $**p \leq 0.01$, $***p \leq 0.001$.

FIGURE S4. Harmine does not affect coronavirus genomic, total RNA or replication foci localization. Huh7 cells were infected with HCoV-229E virus (MOI of 2) and treated with DMSO or Harmine at 16 hpi. Cells were fixed immediately (t=0), 4.5 h or 9 h post DMSO or compound addition and probed for **(a)** viral dsRNA and N protein localization or **(b)** viral genomic and total RNA localization. Images were taken by Zeiss microscope with AxioCamICc 5 camera at 63 X oil immersion using Zen software.

FIGURE S5. Validation of DGE by RT-qPCR. A subset of the most differentially expressed genes were validated by RT-qPCR upon **(a)** Harmine or **(b)** 1H3 treatment across 2 to 4 different donors. On the right side of the bar graphs are tables showing fold-changes demonstrated by RNA-Seq.

FIGURE S6. Characterization and Validation of the ASEs upon Harmine or 1H3 treatment.

(a) Pie-charts showing distribution of differentially spliced events at $|\Delta PSI| \geq 10$, adjusted p-value ≤ 0.05 for Harmine vs DMSO (left) and 1H3 vs DMSO (right). **(b)** The left and the right panels are the scatter plots that show changes in the splicing patterns of the genes/events for Harmine vs DMSO and 1H3 vs DMSO treatments, respectively. Scatter plots were generated by R Studio. **(c)**Validation of a subset of perturbed ASEs upon Harmine or 1H3 treatment. PCR amplicons were run on 7-8% polyacrylamide gels and imaged on Chemidoc MP Imager (Bio-Rad). Below each gel image is the respective quantitation (in %) of exon inclusion over total bands in each lane using Bio-Rad ImageLab software.

FIGURE S7. Characterization of the ASEs by gene ontology enrichment analysis and manual curation of data.

(a) Gene ontology enrichment analysis of differentially spliced events/genes upon Harmine treatment, using gProfiler. Below it is the table listing the genes that enriched for different GO terms **(b)** Bar graphs showing splicing profiles of many host RNA processing factors upon Harmine (top) or 1H3 (bottom) treatment, identified by manual curation of RNA processing as functional annotation.

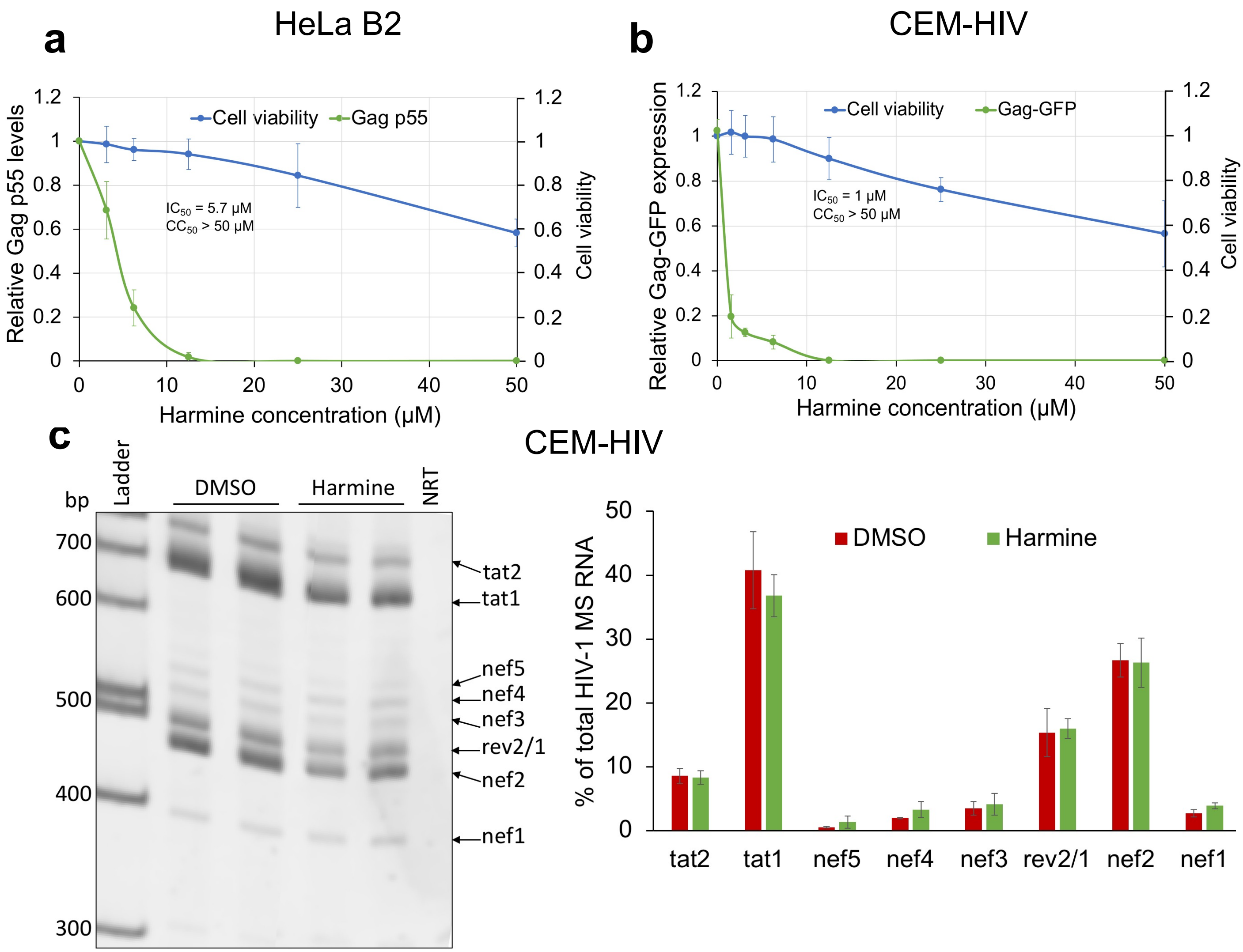
a
HeLa B2
Cell viability
Gag p55
IC50 = 5.7 µM
CC50 > 50 µM
Relative Gag p55 levels
Cell viability
Harmine concentration (µM)
b
CEM-HIV
Cell viability
Gag-GFP
IC50 = 1 µM
CC50 > 50 µM
Relative Gag-GFP expression
Cell viability
Harmine concentration (µM)
c
CEM-HIV
bp
Ladder
DMSO
Harmine
NRT
700
600
500
400
300
tat2
tat1
nef5
nef4
nef3
rev2/1
nef2
nef1
% of total HIV-1 MS RNA
DMSO
Harmine
tat2
tat1
nef5
nef4
nef3
rev2/1
nef2
nef1

Figure S1

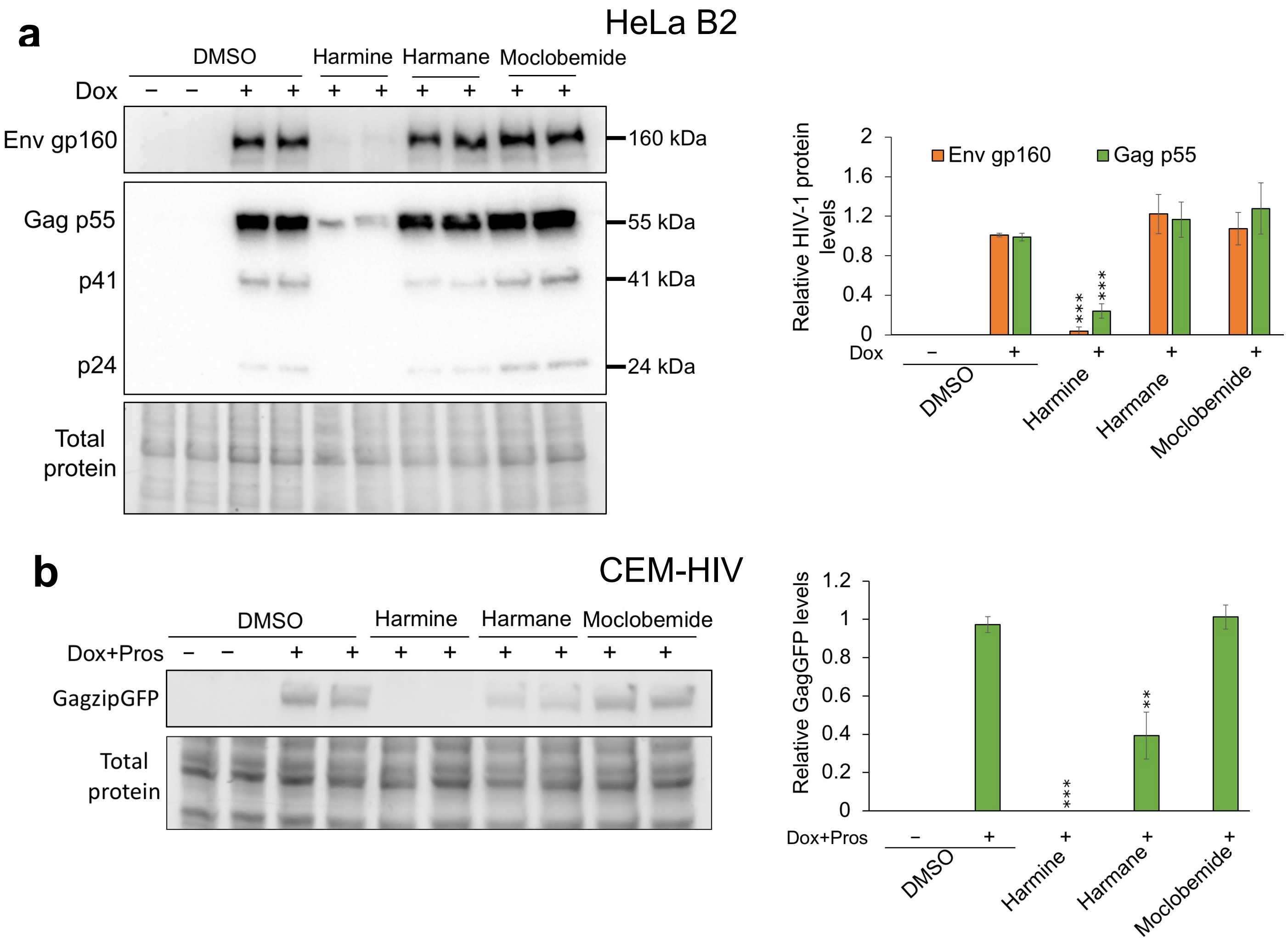
a
HeLa B2
DMSO
Harmine
Harmane
Moclobemide
Dox
Env gp160
Gag p55
p41
p24
Total protein
160 kDa
55 kDa
41 kDa
24 kDa
Relative HIV-1 protein levels
Env gp160
Gag p55
2
1.6
1.2
0.8
0.4
0
***
***
b
CEM-HIV
Dox+Pros
GagzipGFP
Relative GagGFP levels
1.2
1
0.8
0.6
0.4
0.2
0
**

Figure S2

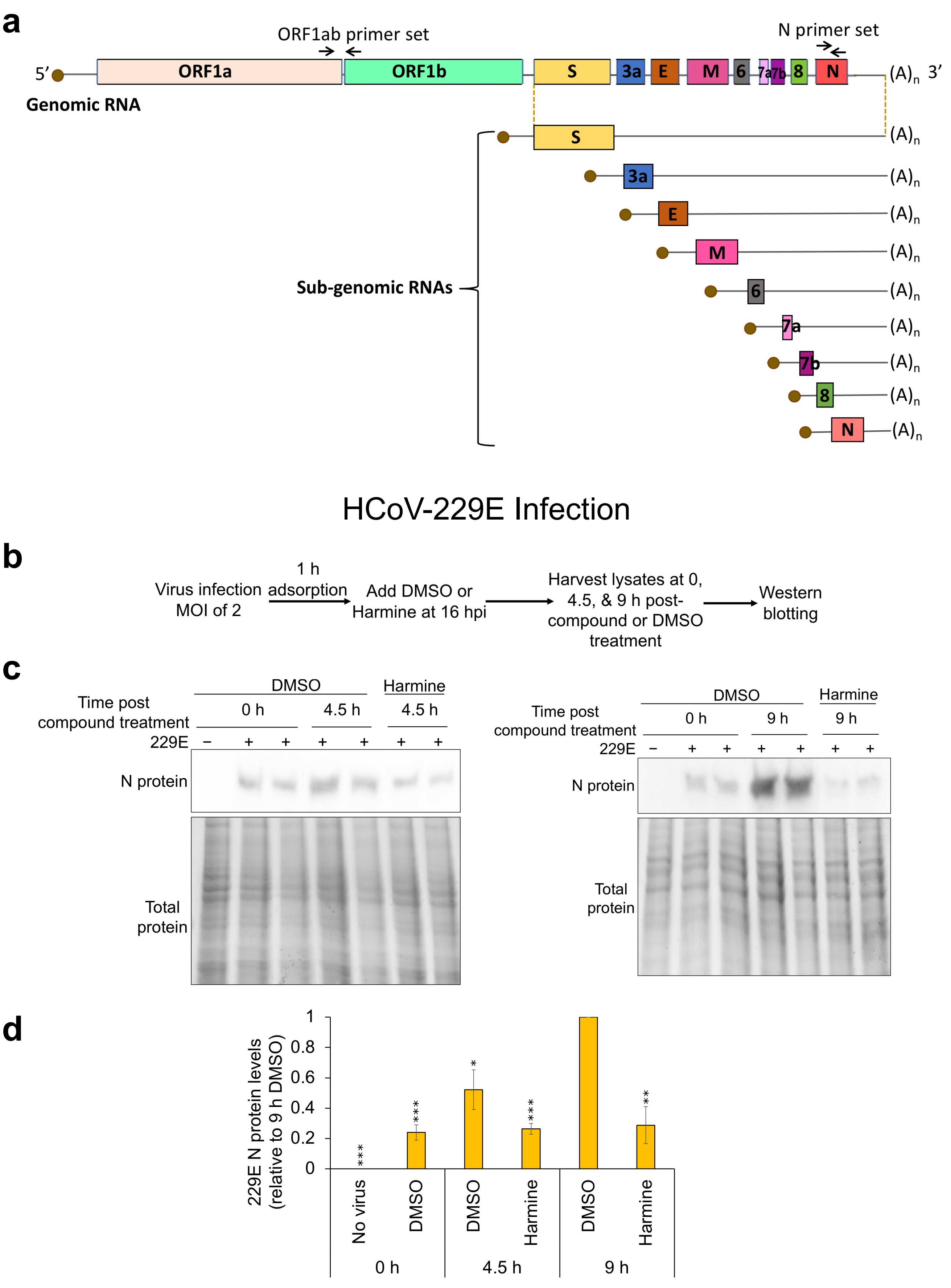
a
ORF1ab primer set
N primer set
5′
ORF1a
ORF1b
S
3a
E
M
6
7a
7b
8
N
(A)n 3′
Genomic RNA
Sub-genomic RNAs
HCoV-229E Infection
b
Virus infection MOI of 2
1 h adsorption
Add DMSO or Harmine at 16 hpi
Harvest lysates at 0, 4.5, & 9 h post-compound or DMSO treatment
Western blotting
c
DMSO
Harmine
Time post compound treatment
0 h
4.5 h
9 h
229E
N protein
Total protein
d
229E N protein levels (relative to 9 h DMSO)
1
0.8
0.6
0.4
0.2
0
No virus
DMSO
Harmine
0 h
4.5 h
9 h

Figure S3

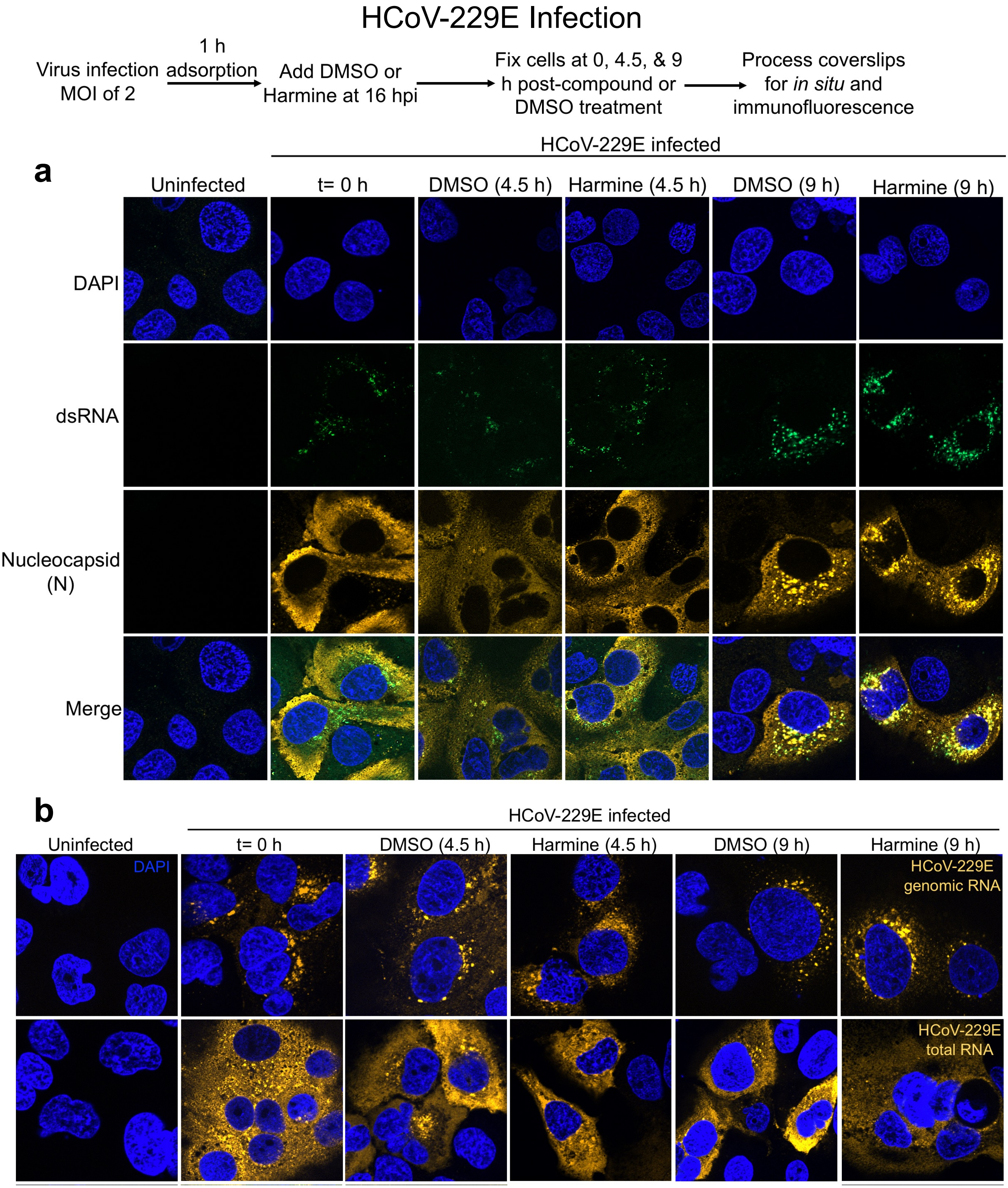
HCoV-229E Infection
Virus infection MOI of 2
1 h adsorption
Add DMSO or Harmine at 16 hpi
Fix cells at 0, 4.5, & 9 h post-compound or DMSO treatment
Process coverslips for in situ and immunofluorescence
a
HCoV-229E infected
Uninfected
t= 0 h
DMSO (4.5 h)
Harmine (4.5 h)
DMSO (9 h)
Harmine (9 h)
DAPI
dsRNA
Nucleocapsid (N)
Merge
b
HCoV-229E infected
Uninfected
t= 0 h
DMSO (4.5 h)
Harmine (4.5 h)
DMSO (9 h)
Harmine (9 h)
DAPI
HCoV-229E genomic RNA
HCoV-229E total RNA

Figure S4

a

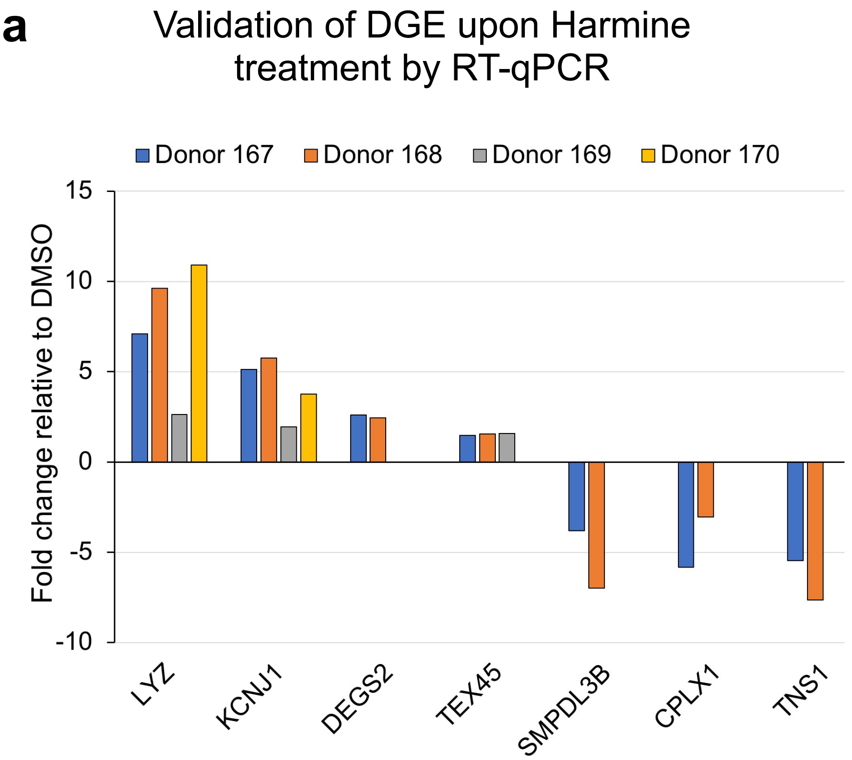

Fold change demonstrated by RNA-Seq data

Genes	Log_2fold change(LFC) observed in RNA-Seq	LFC conversion to fold-change
LYZ	4.0176	16.196
KCNJ	2.9371	7.66
DEGS2	5.3008	39.42
TEX45	3.5363	11.6
SMPDL3B	-2.8043	0.14
CPLX1	-3.0662	0.119
TNS1	-3.1707	0.111

b

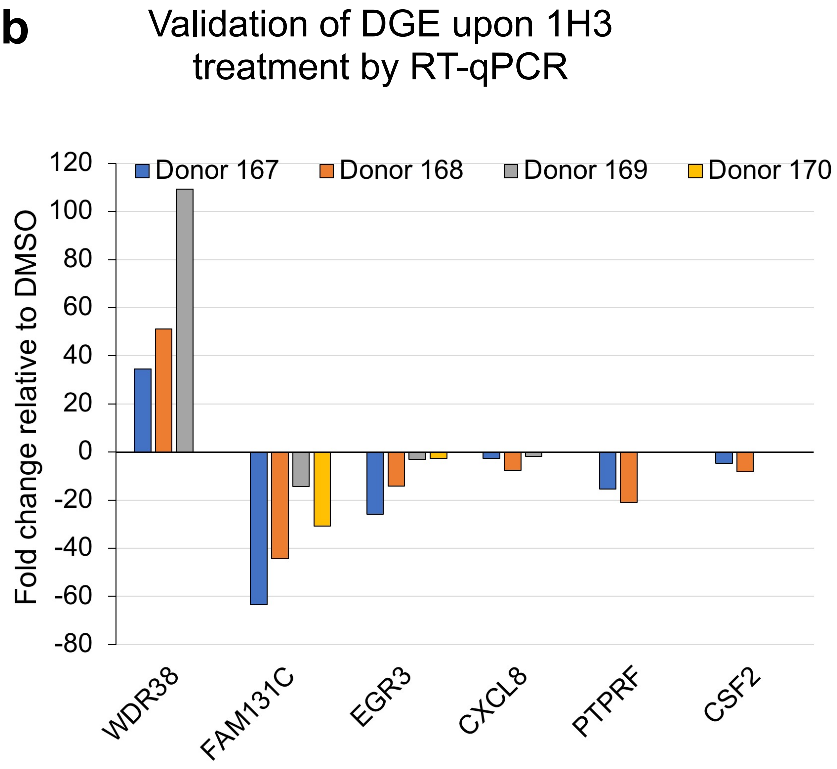

Fold change demonstrated by RNA-Seq data

Genes	Log_2fold change(LFC) observed in RNA-Seq	LFC conversion to fold-change
WDR38	9.9722	1004
FAM131C	-5.7533	0.018
EGR3	-5.2109	0.027
CXCL8	-5.649	0.020
PTPRF	-5.3029	0.025
CSF2	-5.7177	0.019

Figure S5

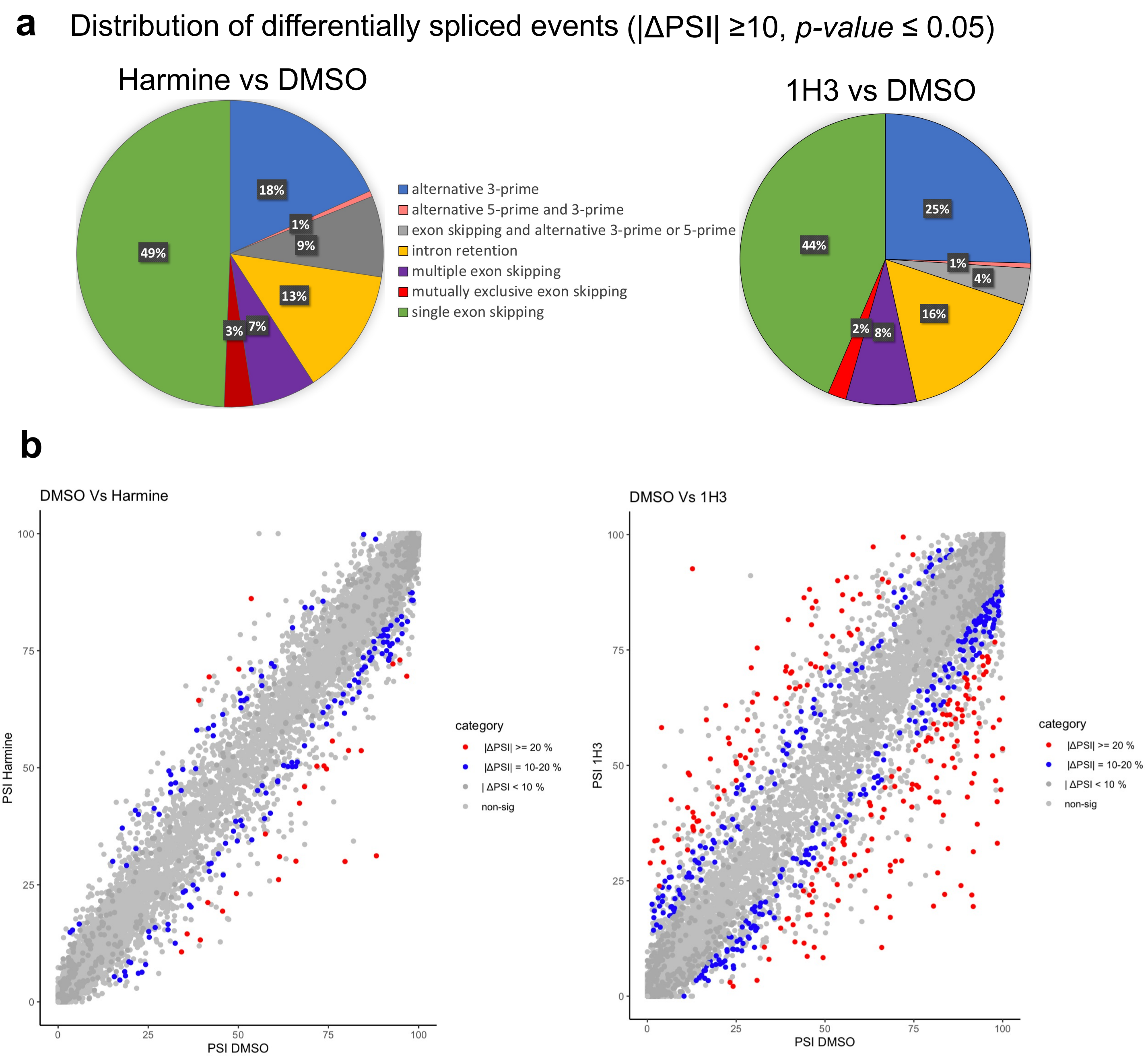
a
Distribution of differentially spliced events (|ΔPSI| ≥10, p-value ≤ 0.05)
Harmine vs DMSO
18%
1%
9%
13%
7%
3%
49%
alternative 3-prime
alternative 5-prime and 3-prime
exon skipping and alternative 3-prime or 5-prime
intron retention
multiple exon skipping
mutually exclusive exon skipping
single exon skipping
1H3 vs DMSO
25%
1%
4%
16%
8%
2%
44%
b
DMSO Vs Harmine
PSI Harmine
PSI DMSO
category
|ΔPSI| >= 20 %
|ΔPSI| = 10-20 %
| ΔPSI < 10 %
non-sig
DMSO Vs 1H3
PSI 1H3
PSI DMSO
category
|ΔPSI| >= 20 %
|ΔPSI| = 10-20 %
| ΔPSI < 10 %
non-sig

Figure S6

c

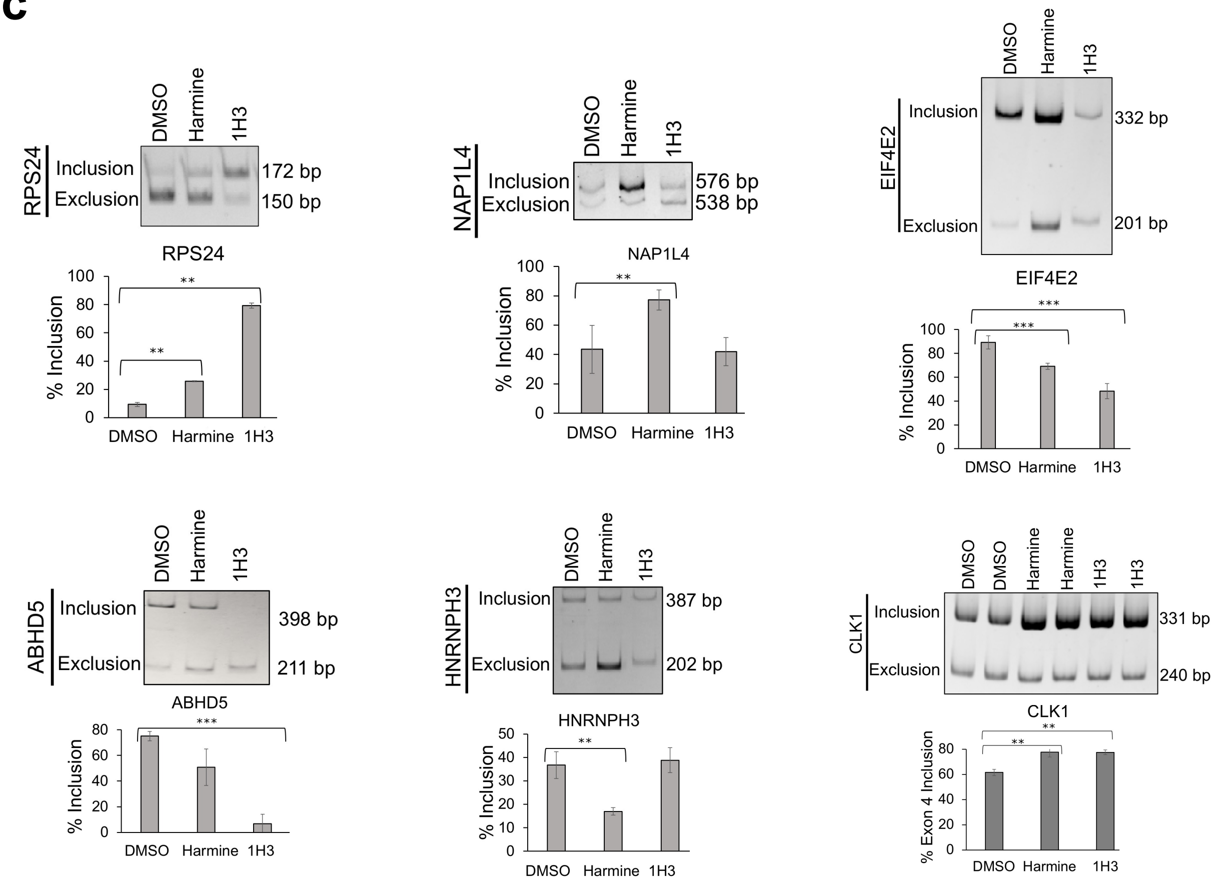
RPS24
DMSO
Harmine
1H3
Inclusion
Exclusion
172 bp
150 bp
% Inclusion
**
NAP1L4
576 bp
538 bp
EIF4E2
332 bp
201 bp
***
ABHD5
398 bp
211 bp
HNRNPH3
387 bp
202 bp
CLK1
331 bp
240 bp
% Exon 4 Inclusion

a Gene ontology (GO) enrichment analysis of differentially spliced events/genes upon Harmine treatment

Term Name	Term ID	P adj.	$-\log_{10}$(P adj.)
regulation of I-kappaB kinase/NF-kappaB signaling	GO:0043122	1.202×10^{-3}	
regulation of RNA stability	GO:0043487	3.350×10^{-2}	
cellular response to stress	GO:0033554	4.574×10^{-2}	

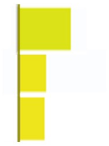

Term Name	Genes
regulation of I-kappB kinase/NF-kappaB signaling	CARD19,CARD8,CFLAR,DHX36,EEF1D,FLNA,FYN,GAPDH,MYD88,RHOH,TNIP1
regulation of RNA stability	DHX36,EXOSC2,EXOSC3,MAPKAPK2,MYD88,PABPC4,TBRG4,TENT2
cellular response to stress	BCLAF1,BRD8,CCM2,CFLAR,DCLRE1C,DHX36,DYRK2,EHMT2,FAM111A,FLNA,FYN,HMGA1,IFFO1,IL18BP,KAT5,KMT5B,LANCL1,MAPKAPK2,MYD88,NR1H2,NUDT1,PTPN2,RBBP5,RNF185,SLC11A2,SLC38A2,TP53BP1,XPC

https://biit.cs.ut.ee/gprofiler/gost

b Splicing profiles of host RNA processing factors

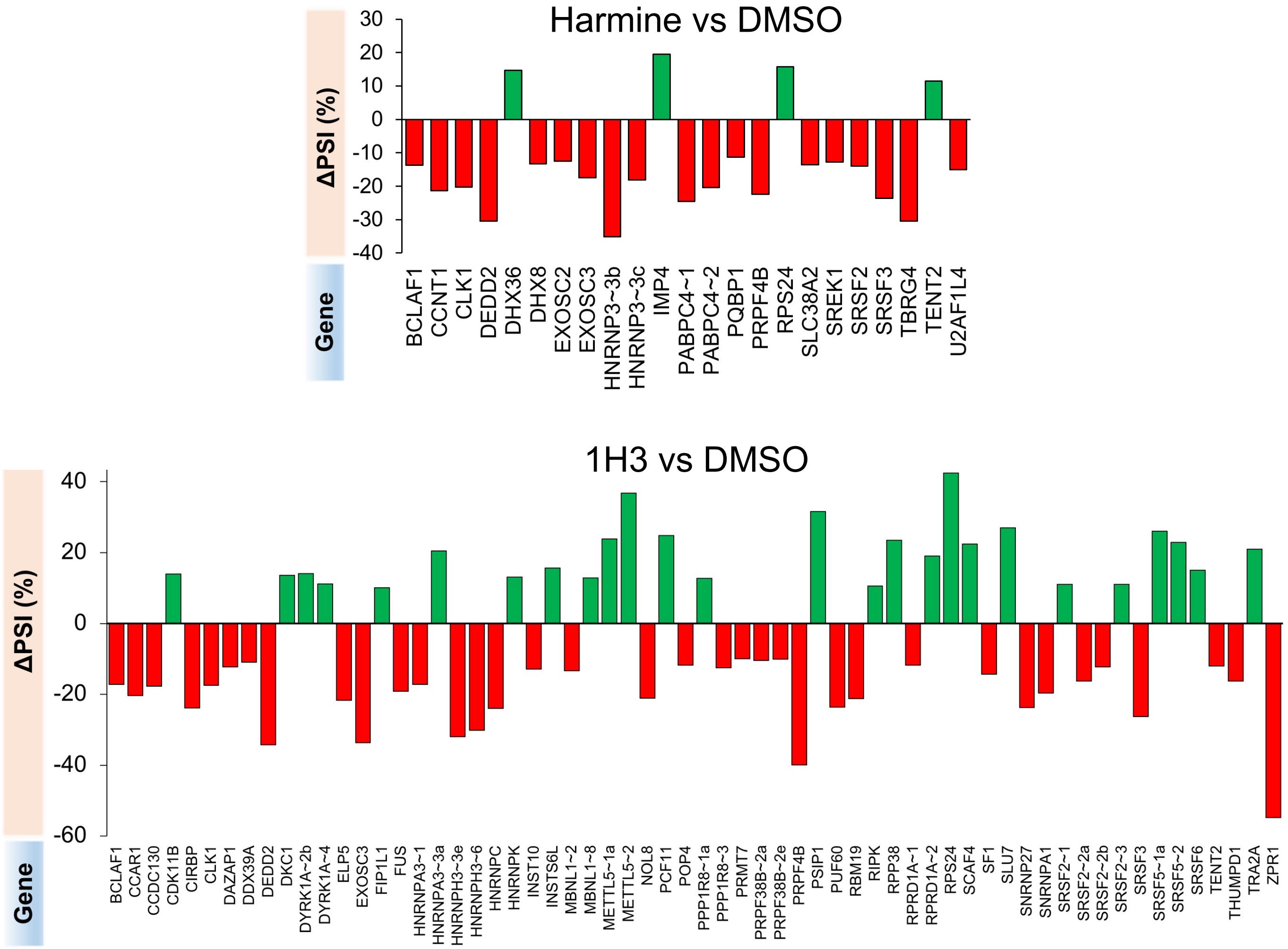

Figure S7
